# Design of Lytic Phage Cocktails Targeting *Salmonella*: Synergistic Effects Based on In Vitro Lysis, In Vivo Protection, and Biofilm Intervention

**DOI:** 10.3390/v17101363

**Published:** 2025-10-12

**Authors:** Mengrui Zhang, Qishan Song, Zhengjie Liu, Martha R. J. Clokie, Thomas Sicheritz-Pontén, Bent Petersen, Xiaoqian Wang, Qing Zhang, Xiaohui Xu, Yanbo Luo, Pingbin Lv, Yuqing Liu, Lulu Li

**Affiliations:** 1 Key Laboratory of Livestock and Poultry Multi-Omics of MARA, Institute of Animal Science and Veterinary Medicine, Shandong Academy of Agricultural Sciences, Jinan 250100, China; sandyzmr@outlook.com (M.Z.); zhangqingchina@163.com (Q.Z.); xmsxuxiaohui@163.com (X.X.); luoyanbosaas@163.com (Y.L.); liuiuqing@163.com (Y.L.); 2 China-UK Joint Laboratory of Phage Engineering, Sino-Danish Joint Laboratory of Microbial Informatics, Jinan 250100, China; zl271@leicester.ac.uk (Z.L.); mrjc1@leicester.ac.uk (M.R.J.C.); thomassp@sund.ku.dk (T.S.-P.); bent.petersen@sund.ku.dk (B.P.); 3 Shanghe County Center for Animal Husbandry and Veterinary Development, Jinan 251600, China; songqishan73@foxmail.com (Q.S.); shangheyikong@jn.shandong.cn (P.L.); 4 Becky Mayer Centre for Phage Research, University of Leicester, Leicester LE1 7RH, UK; 5 Center for Evolutionary Hologenomics, University of Copenhagen, 1165 Copenhagen, Denmark; 6 Centre of Excellence for Omics-Driven Computational Biodiscovery (COMBio), Faculty of Applied Sciences, AIMST University, Bedong 08100, Malaysia; 7 College of Veterinary Medicine, Institute of Comparative Medicine, Yangzhou University, Yangzhou 225009, China; wxq13616361750@163.com

**Keywords:** bacteriophage, phage cocktail, *Salmonella* Typhimurium, *Salmonella* Enteritidis, *Galleria mellonella*, biofilm

## Abstract

*Salmonella* is a major zoonotic pathogen and phage cocktails offer a novel strategy against its infections. This study aimed to characterize *Salmonella* phages and assess the efficacy of various phage combinations, both in vitro and in vivo. Three phages (PJN012, PJN042, PJN065) were isolated, showing stability across a broad range of temperatures and pH values, and lacking genes associated with lysogenicity, virulence, and antibiotic resistance. Combined with two known phages (PJN025, vB_SalS_JNS02), they formed cocktails tested for lytic activity against *S*. Enteritidis and *S*. Typhimurium. Phage cocktails (comprising 2–5 phages) that demonstrated efficacy in vitro were validated using *Galleria mellonella* models. For *S*. Enteritidis strain 015, prophylactic cocktail C18 increased larval survival to 90% at 48 h (vs. 3% control). For *S*. Typhimurium strain 024, phage cocktail 26 showed the best therapeutic effect when co-injected with the bacterium, with a survival rate of up to 85% at 96 h, compared to 30% in the positive control group. Biofilm assays showed cocktails inhibited formation more effectively (e.g., at 24 h, C14 and C17 reduced biofilm formation by 93.74% and 94.21%, respectively) than removed established ones. The cocktails depended on bacterial type, phage genera, combinations, and incubation time. Robust in vitro screening remains crucial for optimizing phage formulations despite potential in vivo discrepancies.

## 1. Introduction

*Salmonella*, a ubiquitous foodborne pathogen with global implications, poses a significant threat to livestock production, food safety, and public health worldwide [1,2,3]. Comprising *S. enterica* and *S. bongori*, this genus comprises an extensive array of over 2600 serotypes. Traditionally, salmonellosis control has relied heavily on antibiotics; however, this intensive use has inadvertently fostered the emergence of multidrug-resistant *Salmonella* strains. Consequently, exploring alternative strategies, such as the utilization of bacteriophages, has become increasingly important as a potential method for reducing bacterial contamination.

In practice, phage therapy primarily takes two forms: monophage therapy and phage cocktail (multiphage) therapy. However, individual phages often have a narrow host range limited to a subset of bacterial strains, significantly restricting their efficacy in controlling diverse bacterial infections. Moreover, relying solely on a single phage type can accelerate the emergence of phage-resistant bacterial variants [4,5]. Recognizing these limitations, the concept of phage cocktails has emerged, involving the strategic combination of multiple diverse phages. This approach aims to broaden the host range, reduce the risk of phage resistance, and enhance overall efficacy against bacterial infections. Recent studies have adopted this strategy by formulating phage cocktails tailored to target a broad spectrum of *Salmonella* serovars. These cocktails have shown promising results in reducing *Salmonella* colonization in animals [6,7,8,9] and meat products [10,11,12], as well as controlling *Salmonella* biofilms [13,14] and contamination in food manufacturing environments [15].

Biofilms have received significant global health attention, as they are implicated in the majority of bacterial infections due to their ability to enhance microbial resilience against external stressors, particularly antibiotics [16]. *Salmonella* species are notably persistent in the environment and can form complex surface-attached communities, known as biofilms, on food surfaces [17]. According to the Centers for Disease Control and Prevention, biofilms are responsible for approximately 80% of bacterial diseases [18]. Consequently, it is imperative to identify alternative solutions to antimicrobials that effectively inhibit biofilm. Notably, several studies have documented the inhibition of *Salmonella* biofilms by bacteriophages [19,20,21]. These investigations have explored the inhibitory effects of diverse phage cocktails with varying titers on multiple surfaces, such as 96-well plates, stainless steel surfaces, poultry drinking systems, and ready-to-consume food products. However, these studies have focused solely on endpoint analysis rather than kinetic assessments. Furthermore, research has predominantly emphasized the ability of phages either to prevent biofilm formation or to eradicate established biofilms.

Although numerous studies have reported successful applications of phage cocktails, these cocktails are frequently assembled through random combinations, and most experiments have only evaluated the efficacy of fixed cocktail compositions. Such random assembly often leads to unstable lytic effects, limiting their practical application against specific pathogens. Researchers generally believe that the lytic effect of a phage cocktail is not linearly positively correlated with the number of phages it contains. Sometimes, the introduction of an excessive number of different phage types may instead trigger competitive inhibition among phages (e.g., competing for binding sites), thereby impairing the overall lytic effect [22,23]. This finding serves to reinforce the notion that rational phage cocktail design, beyond the selection of optimal phage types, must additionally incorporate the optimization of phage numbers to avoid inter-phage competition.

The objective of our study was to develop an effective phage cocktail without the need for a detailed analysis of phage receptors. Five bacteriophages, three newly isolated and two previously characterized, were combined in various combinations to create candidate phage cocktails. The inhibitory effects of different cocktail formulations against *S*. Enteritidis and *S*. Typhimurium were evaluated in liquid cultures. Cocktails consisting of two, three, four, or five phages, which exhibited superior lytic activity, were subsequently evaluated in vivo for their potential therapeutic efficacy. Additionally, we investigated the efficacy of various phage cocktails in preventing biofilm formation and eradicating preformed biofilms over multiple periods.

## 2. Materials and Methods

### 2.1. Bacterial Host Characterization

#### 2.1.1. Bacterial Strains

All bacterial strains (Supplementary Table S1) used in this study were maintained in our laboratory for the specific purpose of phage isolation and lytic spectrum evaluation. Notably, the *Salmonella* isolates were obtained from chickens, ducks, and pigs collected from various provinces across China.

#### 2.1.2. Assessment of Biofilm Forming Ability

30 *Salmonella* strains, comprising 17 strains of *S*. Enteritidis and 13 of *S*. Typhimurium, were selected, and their ability to form biofilms was assessed. Biofilm formations evaluated using a modified crystal violet assay, as previously described by Islam et al. [20]. Overnight cultures were diluted in LB broth without NaCl to a standardized concentration of 10^8^ CFU/mL. Aliquots of 200 µL from each diluted sample were dispensed in triplicate into 96-well microplates. These plates were incubated at 37 °C for 24, 48, and 72 h, with the medium renewed every 24 h to observe and analyze biofilm formation over time.

After each incubation period, the contents of the wells were aspirated, and the plates were rinsed three times with PBS (pH 7.2) to eliminate any nonadherent bacterial cells. To fix the remaining adherent cells, 200 µL of 98% methanol was added to each well and incubated for 15 min. After discarding the methanol and allowing the plates to air dry, 200 µL of 1% crystal violet dye was added to each well and incubated for 30 min to stain the biofilm. Excess dye was gently rinsed off with tap water, after which 200 µL of 33% glacial acetic acid was added to each well. This acetic acid solution was left for 30 min to facilitate solubilization of the crystal violet-stained biofilm. Biofilm formation was quantified using a microplate photometer set to a wavelength of 595 nm. Biofilm production was classified into four categories, namely, negative, weak, moderate, and strong, based on predetermined cutoff values [13].

### 2.2. Isolation and Propagation of Bacteriophages

Bacteriophages targeting *Salmonella* were isolated from a variety of sources, including sewage, fecal matter, and other environmental samples collected throughout Shandong Province using the standard double-layer agar (DLA) technique. To concentrate the phage suspensions, the isolated phages were treated with polyethylene glycol 8000 (PEG 8000, Sigma Aldrich, Louis, MO 63103, USA). This was followed by a rigorous purification step through CsCl density gradient ultracentrifugation with three calibrated CsCl solutions at densities of 1.7, 1.5, and 1.3 g/mL [24]. The concentrated and partially purified phage particles were then dialyzed in PBS buffer (0.1 M, pH 7.4) to remove excess salts and impurities. To further purify the phages and ensure suitability for downstream applications, the dialyzed suspension was passed through Detoxi-Gel (Pierce Biotechnology, Rockford, IL, USA), an endotoxin-removing gel, three times. This additional purification step effectively eliminated any potential endotoxins present in the phage preparation. Finally, the highly purified phage suspension was serially diluted and re-evaluated using the DLA method to accurately determine the phage titer.

### 2.3. Characterization of Bacteriophages

#### 2.3.1. Host Range Determination

The host range of the isolated phages was evaluated via the spot assay [25] against a total of 149 bacterial strains. This strain panel comprised 32 *S*. Enteritidis, 74 *S*. Typhimurium, 3 *S*. Pullorum, and 10 strains of *Escherichia coli*, *Klebsiella pneumoniae*, *Enterococcus* spp., and *Pseudomonas aeruginosa*. The spot assay was specifically utilized to assess the bactericidal potential of all isolated phages. Following incubation at 37 °C for 14–16 h, the plates were inspected for signs of bacterial lysis. A host strain was defined as sensitive if individual plaques were observable. All phage-strain combinations were tested in at least two independent experiments to ensure reproducibility of results.

#### 2.3.2. Transmission Electron Microscopy (TEM) Analysis

A 10 µL aliquot of the bacteriophage suspension was applied to a carbon-coated grid with nicks and allowed to adsorb for 5 min. Excess suspension was then removed by rinsing with sterile distilled water. The grid was air-dried for an additional 5 min and negatively stained with a 2% solution of uranyl acetate. Transmission electron microscopy was performed using a JEM-1400 FLASH TEM (JEOL Ltd., Tokyo, Japan).

#### 2.3.3. Temperature and pH Stability Assay

Individual phage stability was determined through the incubation of phage particles suspended in SM buffer under varying temperature and pH settings. Specifically, for the temperature stability assay, phages were incubated at 30, 40, 50, 60, 70, and 80 °C for two distinct durations (30 and 60 min), after which their titers were determined using the DLA method. For the pH stability assay, the phage suspension was mixed at a 1:10 volume ratio with SM buffer pre-adjusted to pH values spanning 1 to 13. Following a 2 h incubation at 37 °C, the phage titer was measured via the DLA method. All experiments were conducted in triplicate to ensure reproducibility.

#### 2.3.4. Determination of Optimum Multiplicity of Infection (MOI)

A 100 µL aliquot of bacterial culture (~10^5^ CFU/mL) was thoroughly mixed with an equal amount (100 µL) of phage suspension prepared in SM buffer across various multiplicities of infection (MOIs) ranging from 0.001 to 1000. Subsequently, 2 mL of LB broth was added to the mixture. The samples were incubated at 37 °C with agitation 2 h to facilitate phage-bacteria interactions. Phage lysates were then titrated to identify the optimal MOI, defined as the condition that yielded the highest phage titer. All experiments were performed in triplicate to ensure the reproducibility and reliability of the results.

#### 2.3.5. One-Step Growth Assay

The methodology described by Kropinski [26] was followed with strategic modifications. Cultures of *Salmonella* strains (10^5^ CFU/mL) were combined with phages at an MOI of 100. This mixture was incubated at 37 °C for 5 min to allow efficient phage absorption. To eliminate unbound phages, the mixture was subsequently centrifuged at 4200× *g* for 10 min. The resulting bacterial pellet was resuspended in LB broth and incubated at 37 °C with agitation to facilitate phage replication and release. Aliquots were collected every 10 min over a 2 h period, and phage titers were determined using the DLA method. All experiments performed in triplicate.

#### 2.3.6. Phage DNA Extraction, Sequencing, and Analysis

Phage DNA was extracted using the standard protocol provided by the Phage Genome DNA Quick Extraction Kit (Beijing Zhuangmeng International Biology Gene Technology Co., Ltd., Beijing, China). Prior to sequencing on the Illumina HiSeq platform, DNA quality was assessed using a NanoDrop 2000 spectrophotometer, and quantity was measured with the Qubit dsDNA HS Assay Kit on a Qubit fluorometer (Thermo Scientific, San Diego, CA, USA). Gene prediction was performed using the Rapid Annotation Using Subsystem Technology (RAST) server [27]. To analyze sequence similarity, we performed using the Basic Local Alignment Search Tool (BLAST) from the National Center for Biotechnology Information’s (NCBI) (http://blast.ncbi.nlm.nih.gov/, 10 July 2023). Potential antibiotic resistance and virulence factors were identified using the Antibiotic Resistance Database (ARDB, https://card.mcmaster.ca/analyze/rgi, 15 July 2023) and the Virulence Factor Database (VFDB, http://www.mgc.ac.cn/VFs/, 16 July 2023), respectively. tRNA genes were identified using tRNAscan-SE [28]. Phage genome architecture was visualized using the Proksee online platform (https://proksee.ca/, 21 July 2023) [29], providing a comprehensive gene map. A phylogenetic tree of the five phages was constructed using ViPTree version 4.0 (http://www.genome.jp/viptree/, 27 July 2023) [30].

### 2.4. Preparation of Phage Cocktails

Twenty-six distinct phage cocktails were prepared through random permutations of the bacteriophages, comprising combinations of two, three, four, or all five phages (as detailed in Table 1). During formulation, all phages were maintained at the same concentration and mixed in equal volumes, ensuring that the final concentration of each phage was consistent across all cocktails.

#### 2.4.1. Testing Efficacy of Phage Cocktails

The *Galleria mellonella* larva model is especially valuable for avoiding ethical concerns and cost-related issues associated with animal use, and it has been employed previously to investigate the pathogenesis and virulence of diverse pathogens [31,32]. To establish an effective infection model using *G. mellonella*, we first conducted challenge experiments with meticulously selected strains of *Salmonella*, applying a standardized challenge dose across all candidates. From this initial screening, we identified the strain that presented the highest mortality rate. Ultimately, two strains were selected as optimal candidates: *S*. Enteritidis strain SSCZ18090015 (hereafter referred to as 015) and the *S*. Typhimurium strain SSDZ1810024 (hereafter referred to as 024), which were used in subsequent phage cocktail efficacy experiment. In these experiments, phages and phage cocktails were administered at an MOI of 100.

#### 2.4.2. Planktonic Killing Assay (PKA)

PKA was systematically performed using both individual phages and various phage cocktails. Specifically, 100 µL of 015 or 024 (10^5^ CFU/mL) was added to each well of a 96-well microplate. An equal volume (100 µL) of each individual phage or cocktail was then added to the designated wells. A mixture of 100 µL of bacteria (10^5^ CFU/mL) with 100 µL of LB broth was used as the positive control, while 200 µL of LB broth served as the negative control. Bacterial turbidity was measured every 30 min over a 24 h period using a spectrophotometer at a wavelength of 600 nm (OD_600_). Data were expressed as the mean value from triplicate measurements conducted in two separate independent experiments. The curves representing the killing assay were analyzed using a modified objective method specifically devised, employing the generated curve to derive a “virulence index” [33]. The virulence index, a key metric defined to quantify the virulence of a specific phage against its host, can be categorized into four distinct grades based on its numerical values. Specifically, these grades are classified as follows: high virulence, corresponding to a virulence index score greater than 0.5; medium virulence, with a virulence index score ranging from 0.2 to 0.5; low virulence, where the virulence index score is between ≥0.001 and <0.2; and no virulence, indicated by a virulence index score of 0.

#### 2.4.3. Phage Cocktail Therapy for *Salmonella* Infection in G. mellonella

In preliminary experiments, we determined the LD_50_ of two *Salmonella* strains (015 and 024) to be 10^6^ CFU/mL and 10^5^ CFU/mL, respectively. Guided by PKA assays, we further screened effective phage combinations against each strain, constructing 4 phage cocktails per strain (each comprising 2, 3, 4, or 5 phages). To validate the efficacy of these cocktails against *Salmonella* infection, we conducted independent experiments on each strain using *G. mellonella*, with a consistent set of 18 experimental groups: 1 negative control (no challenge, no phage treatment), 1 positive control (infection only), 4 phage groups (treatment with the 4 cocktails respectively, no infection), 4 prophylactic groups, 4 co-infection groups, and 4 remedial groups. For each strain, 360 larvae were randomly assigned to 18 groups [25,34], thereby guaranteeing the reliability and comparability of results.

In the experiments, *Salmonella* strains (at their respective LD_50_ concentrations) and phage cocktails (prepared by mixing phages in equal volumes) were administered to larvae via proleg injection: bacteria were injected into the hindmost left proleg, while phages were injected into the hindmost right proleg. Phage cocktails were administered following three regimens: prophylactically (1 h prior to bacterial challenge), simultaneously with bacteria (co-infection), or as a remedial treatment (1 h after bacterial challenge). After administering bacteria and phages, all larvae were incubated at 37 °C. Larvae were considered deceased if they showed no response to touch and exhibited a color change from light brown to black. All of the larval experiments were performed in triplicate.

### 2.5. Assessment of Bacteriophage Cocktails on Biofilms

An initial screening of thirty *Salmonella* strains was conducted to assess their biofilm-forming capabilities, resulting in the selective inclusion of one strain of *S*. Enteritidis and one strain of *S*. Typhimurium for further investigation. The current section presents an independent analysis of the effects of different cocktails on the control and removal efficacy of *Salmonella* strains.

#### 2.5.1. Evaluation of the Efficiency on Preventing the Biofilm Formation

A standardized overnight culture of *Salmonella* (10^9^ CFU/mL) was diluted in LB broth without NaCl to achieve a final concentration of 10^8^ CFU/mL. Then, 100 µL of this bacterial suspension was dispensed into each well of a 96-well microplate. To these wells, 100 µL of various bacteriophage cocktails (10^9^ PFU/mL) was added, and the mixture was incubated at 37 °C without agitation for 24, 48 and 72 h, respectively. As part of the experimental setup, a positive control well received 100 µL of bacterial culture (10^8^ CFU/mL) mixed with 100 µL of sterile LB broth without NaCl, whereas a negative control well contained only 200 µL of sterile LB broth without NaCl. Following phage treatment, the wells were processed utilizing the crystal violet staining method to assess the biofilm formation and reduction efficacy of the different phage cocktails. The extent of biofilm reduction was quantitatively determined using the following formula [20]:

$$\frac{\left[\left(C-B\right)-\left(T-B\right)\right]}{\left[C-B\right]}\times 100\%$$

#### 2.5.2. Biofilm Eradication

Preliminary experiments indicated that the optimal maturation time for *Salmonella* biofilms was 24 h. A standardized overnight culture of *Salmonella* (20 µL, 10^9^ CFU/mL) was diluted in 180 µL of LB broth without NaCl to achieve a final concentration of 10^8^ CFU/mL, which was subsequently dispensed into the wells of a 96-well microplate. These samples were then incubated at 37 °C for 24 h to allow biofilm formation. After maturation, the bacterial culture was removed, and the established biofilms were washed three times with PBS to remove nonadherent cells. Next, 200 µL of bacteriophage and various phage cocktails, each at a final concentration of 10^9^ PFU/mL, were introduced into the respective wells. The plates were then incubated at 37 °C for 4, 6, 8, 12, 24, and 48 h, to comprehensively evaluate the antibiofilm activity of the phage cocktails over time. At each time point, the quantification of biofilms was performed following previously described procedures, ensuring a logical and seamless progression from biofilm formation to phage treatment and final analysis. The extent of biofilm reduction was quantitatively determined using the formula $\frac{\left[\left(C-B\right)-\left(T-B\right)\right]}{\left[C-B\right]}\times 100\%$ [20], and this calculation provides a percentage reduction in biofilm formation as a result of phage treatment.

### 2.6. Statistical Analysis

Statistical analyses were performed using Prism Software (Version 10.0). The two-way analysis of variance was employed to compare the efficacy of phage cocktails versus individual phages, encompassing their impacts on bacterial growth curves as well as the inhibitory and removal effects of phages on biofilms. The one-way analysis of variance was used to assess differences in phage performance across varying MOI. The Mantel-Cox test was applied to compare the survival rates of *G. mellonella*. All results are presented as mean values, with error bars representing standard deviations (SD). Statistical significance was set at *p* < 0.05, with the following notation: * *p* < 0.05, ** *p* < 0.01, *** *p* < 0.001, and **** *p* < 0.0001.

## 3. Results

### 3.1. Host Bacterial Characterization and Selection of Biofilm-Forming Strains

**Figure 1:**
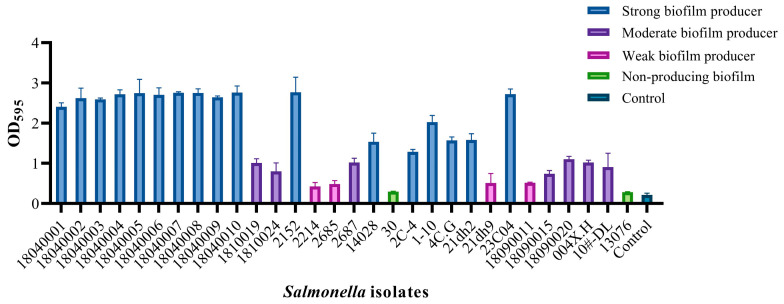
Determination of biofilm-forming ability of 30 *Salmonella* strains for 24 h.

To determine the optimal biofilm maturation time for *Salmonella* strains, OD_595_ value was measured for 30 strains at 24, 48, and 72 h post-incubation. Among these 30 tested strains, 21 exhibited higher OD_595_ values at 24 h compared to those at 48 h and 72 h (Supplementary Figure S1). Based on this observation, the 24 h OD_595_ value was selected as the readout to assess the biofilm-forming ability of the strains. Subsequent quantification results indicated that the 30 strains could be categorized into four distinct groups according to their biofilm-forming capacities: 2 non-biofilm formers, 4 weak biofilm formers, 7 moderate biofilm formers, and 17 strong biofilm formers (Figure 1). To evaluate the efficacy of the bacteriophage cocktail against clinically relevant and robust biofilm producers, two representative strains—*S*. Typhimurium strain 18040005 and *S*. Enteritidis strain 23C04—were selected from the strong biofilm-forming group.

### 3.2. Isolation and Characterization of Bacteriophages

A total of 7 bacteriophages were isolated and named PJN012, PJN042, PJN044, PJN061, PJN065, PJN075, and PJN215.

#### 3.2.1. Determination of Phage Lysis Spectrum

**Figure 2:**
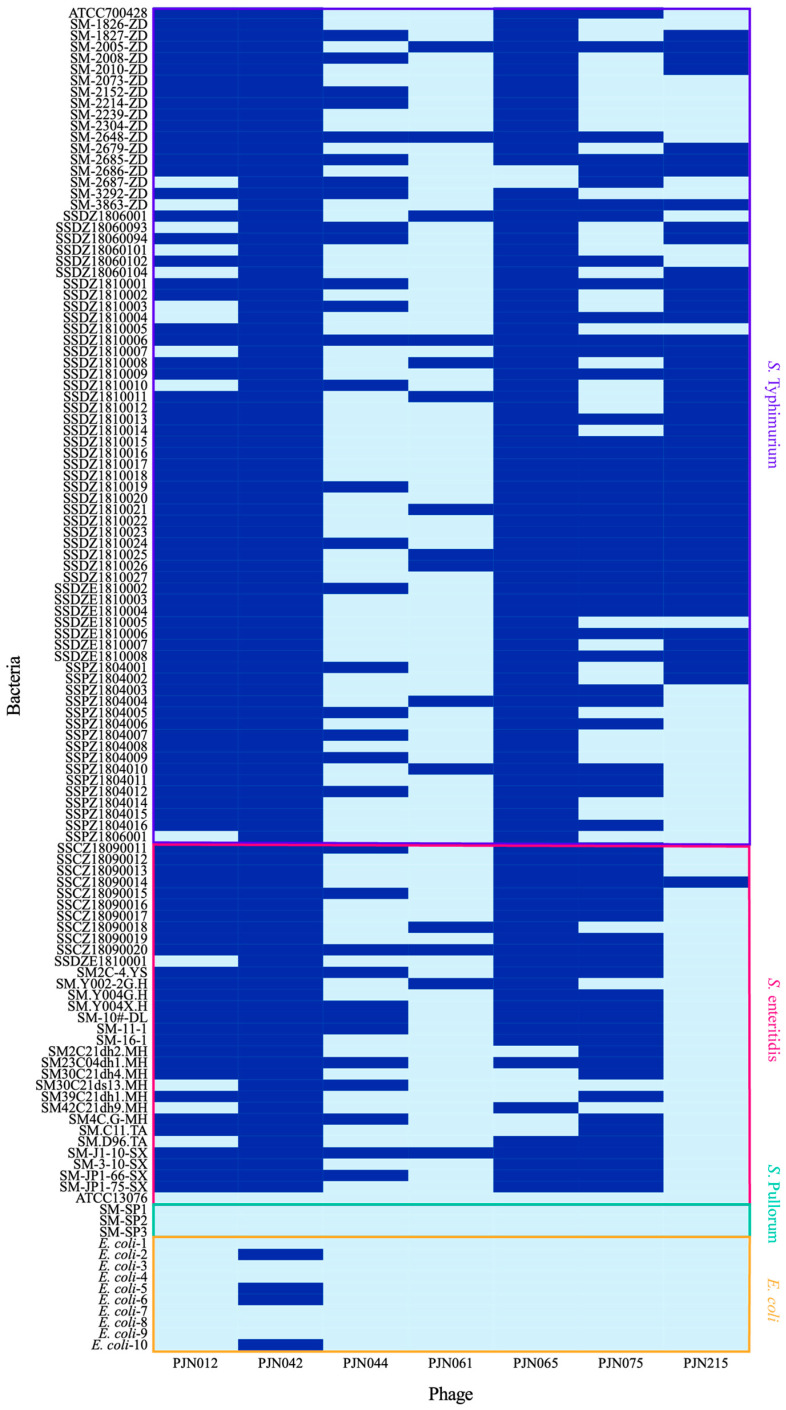
Heatmap visualization of lytic activity of 7 *Salmonella* phages against *S*. Typhimurium (*n* = 74), *S*. Enteritidis (*n* = 32), *S*. Pullorum (*n* = 3), and *Escherichia coli* (*n* = 10): dark blue for lysis (+), light blue for non-lysis (−).

Results of the lytic spectrum assay demonstrated that all 7 phages tested in this study exhibited lytic activity against *S*. Enteritidis and *S*. Typhimurium; however, no lytic activity was detected against the target strains of *S. Pullorum*, *K. pneumoniae*, enterococci, or *P. aeruginosa* included in the experiment. Regarding lytic efficacy against *E. coli*, only phage PJN042 was able to lyse 4 of the tested *E. coli* strains. Figure 2 systematically illustrates the specific strains lysed and the complete lytic spectrum profiles of the aforementioned phages against *S. enteritidis*, *S. typhimurium*, and *E. coli*.

Further analysis of the lysis rate data revealed notable differences in the host ranges of the phages: PJN042 (lysis rate: 73.15%), PJN012 (lysis rate: 66.44%), and PJN065 (lysis rate: 65.10%) displayed a broad host range; PJN075 (lysis rate: 44.97%) and PJN215 (lysis rate: 30.87%) showed a moderately restricted host range; and PJN044 (lysis rate: 22.82%) and PJN061 (lysis rate: 10.07%) exhibited a narrow host range. Based on the aforementioned lysis rate data and the characterization of host range properties, three phages (PJN012, PJN042, and PJN065) were ultimately selected. These phages were combined with two additional phages PJN025 [35] and vB_SalS_JNS02 [25] that had been previously isolated and screened in preliminary experiments to formulate a phage cocktail.

#### 3.2.2. TEM Analysis

**Figure 3:**
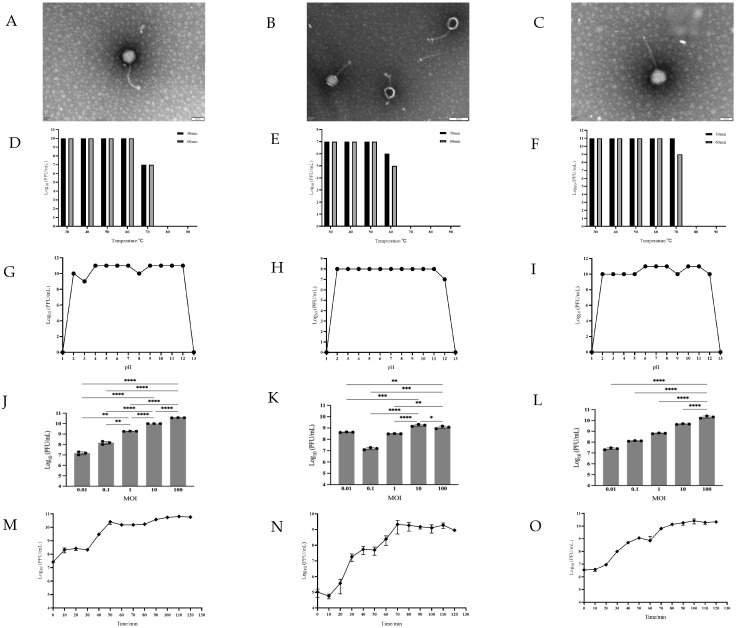
Phenotypical characteristics of the three phages. Panels (**A**–**C**) show morphological characteristics of PJN012 (panel (**A**), white bars 50 nm; magnification ×250,000), PJN042 (panel (**B**), white bars 100 nm; magnification ×200,000), and PJN065 (panel (**C**), white bars 50 nm; magnification ×250,000) under transmission electron micrograph. Panels (**D**–**F**) show the stability of PJN012 (panel (**D**)), PJN042 (panel (**E**)), and PJN065 (panel (**F**)) under different temperature conditions, and panel (**G**–**I**) show the stability of PJN012 (panel (**G**)), PJN042 (panel (**H**)), and PJN065 (panel (**I**)) at different pH values. Panels (**J**–**L**) show the Optimum MOI of PJN012 (panel (**J**)), PJN042 (panel (**K**)), and PJN065 (panel (**L**)), Each point in the figure represents an individual biological replicate. The overall data (e.g., group means) are the average of three independent replicates. Statistical analysis was performed using one-way ANOVA with Tukey’s multiple comparisons test to compare all conditions. Significance levels are indicated as: * *p* ≤ 0.05; ** *p* ≤ 0.01; *** *p* ≤ 0.001; **** *p* ≤ 0.0001, and panels (**M**–**O**) show one-step growth curves of PJN012 (panel (**M**)), PJN042 (panel (**N**)), and PJN065 (panel (**O**)), Data represent the mean of three independent replicates; error bars indicate standard deviation (SD).

Morphological characterization by TEM showed that PJN012, PJN042, and PJN065 had isometric polyhedral heads with long contractile tails ranging from 120 nm to 135 nm (Figure 3A–C).

#### 3.2.3. Thermal and pH Stability of Phages

Thermal stability assessments showed that phages PJN012 and PJN065 remained stable between the temperature range of 30 °C to 60 °C (Figure 3D–F). Specifically, at 70 °C, the titer of PJN012 underwent a 3 log_10_ PFU/mL reduction after both 30 and 60 min of incubation. Conversely, the titer of PJN065 remained unchanged after 30 min but exhibited a 2 log_10_ PFU/mL decline after 60 min. Notably, both phages were completely inactivated at temperatures ranging from 80 °C to 90 °C. In comparison, phage PJN042 maintained a stable titer at temperatures between 30 °C and 50 °C. However, upon incubation at 60 °C, its titer decreased by 1 log_10_ PFU/mL after 30 min and by 2 log_10_ PFU/mL after 60 min. Similarly, PJN042 was rendered completely inactive when exposed to temperatures ranging from 70 °C to 90 °C. These findings underscore the varying degrees of thermotolerance among the phages, with PJN065 exhibiting slightly superior stability at higher temperatures within the tested range.

The results of pH stability showed that these three bacteriophages could survive in the pH 2–12 range (Figure 3G–I). Notably, the titer of PJN042 exhibited remarkable stability, with only a marginal 1 log_10_ PFU/mL decrease observed specifically at pH 12. In contrast, PJN012 displayed a more sensitive response, exhibiting a 1 log_10_ PFU/mL decline in titer at both pH 2 and 8 and a more pronounced 2 log_10_ PFU/mL reduction at pH 3. Intriguingly, PJN065 displayed an unusual trend, with its titer actually increasing by 1 log_10_ PFU/mL within the pH range of 6–8 and 10–11, suggesting a possible increase in its viability under these specific conditions.

#### 3.2.4. Optimum MOI Determination

Under different MOI conditions, the three phages (PJN012, PJN042, and PJN065) exhibited distinct titer profiles. Specifically, PJN012 and PJN065 both achieved their maximum titers at an MOI of 100, reaching 3.7 × 10^10^ PFU/mL and 2.2 × 10^10^ PFU/mL, respectively. Notably, the titers of these two phages at MOI = 100 were significantly higher than those observed under all other MOI conditions (*p* < 0.0001). In contrast, PJN042 displayed its highest titer at an MOI of 10, with a peak value of 1.7 × 10^9^ PFU/mL; and this titer was also significantly higher than the titers of PJN042 under other MOI settings (*p* < 0.05). Based on these results, the optimal MOI values for PJN012, PJN042, and PJN065 were determined to be 100, 10, and 100, respectively (Figure 3J–L).

#### 3.2.5. Result of One-Step Growth Assay

One-step growth curve analysis (Figure 3M–O) revealed a distinctive pattern for each strain. Specifically, the titer of PJN042 initially decreased within the first 10 min, subsequently increased steadily over time, reached a stable plateau phase at approximately 70 min, and ultimately stabilized at 10^10^ PFU/mL. In contrast, both PJN065 and PJN012 exhibited gradual increases in titer from 0 to 10 min. Notably, PJN065 entered its burst period between 20 and 50 min, whereas PJN012 did so between 30 and 50 min. The average burst sizes for PJN012, PJN042, and PJN065 were recorded as 57, 90, and 212 PFU/cell, respectively.

#### 3.2.6. Genomic Analysis

Sequence assembly analysis revealed that PJN012, PJN042, and PJN065 possess double-stranded DNA genomes, comprising 42,557, 111,401, and 42,326 bp, respectively, with corresponding GC contents of 49.60%, 40.15%, and 49.53%, respectively. These comprehensive data were archived in the National Microbiology Data Center (NMDC) under the unique accession numbers NMDCN0004TSM, NMDCN0004TTK, and NMDCN0004TUB, accessible via http://nmdc.cn/resource/genomics/sequence/detail, 1 March 2024.

Comparative analysis against the ARDB and VFDB databases revealed that the genomes of the three phages lacked genes associated with antimicrobial resistance and virulence. Further annotation using the RAST server confirmed that none of the three phage genomes harboured any lysogen-related genes. PJN012, PJN042, and PJN065, contained 62, 161, and 64 open reading frames (ORFs), respectively (Supplementary Tables S2–S4). tRNAscan-SE analysis showed that the PJN042 genome included 22 tRNA genes, including a pseudo-tRNA gene (tRNA^GTA^), which collectively provided at least 18 codons (Supplementary Table S5), while the other two phages did not encode any tRNA genes.

In all three genomes, holin and endolysin genes were located adjacent to each other. In addition to these two functional genes, the PJN042 genome also encodes Rz-like spanin and pyruvate formate-lyase, both of which contribute to host lysis. Analysis of the predicted tail-associated genes revealed variations in their numbers among the phages: PJN012 carries four tail-related proteins, PJN065 has six, and PJN042 has 12. Given that phage tail fibers are intimately linked to phage infection and, subsequently, the host range, this difference may explain why PJN042 has the ability to lyse not only *Salmonella* but also *E. coli*.

**Figure 4:**
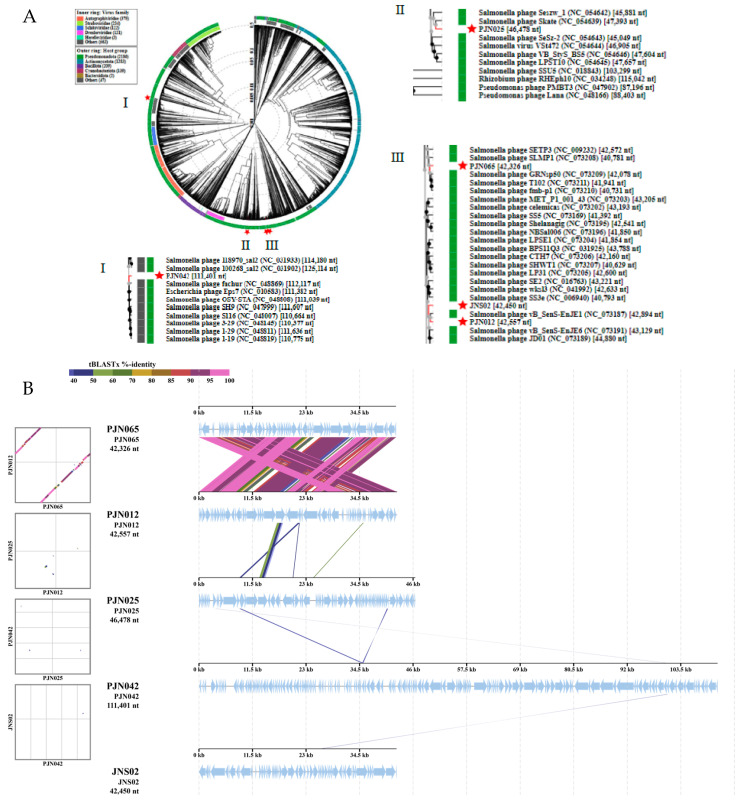
Comparison of five phage genomes. (**A**) Circular proteomic tree based on all predicted CDS using ViPTree version 4.0. (**B**) Genomic alignment of five genomes was analyzed and compared utilizing ViPTree 4.0.

The phylogenetic tree, constructed utilizing all the of predicted CDSs, is depicted in Figure 4A. The three phages, PJN012, PJN065, and JNS02, clearly clustered tightly together because of their shared classification within the *Jerseyvirus* genus. Conversely, the remaining two phages, PJN025 and PJN042, occupy distinct clades, as they belong to the *Skatevirus* and *Epseptimavirus* genera, respectively. By using the Viptree website for comparative analysis, we identified five regions between PJN012 and PJN065 that exhibit a high degree of similarity, exceeding 95% (Figure 4B). In contrast, an ORF found in PJN012 exhibits a degree of similarity to a sequence within the PJN025 genome, ranging from approximately 20% to 60%. Notably, the genomes of the other two bacteriophages, PJN042 and JS02, show minimal resemblance to those of the aforementioned three bacteriophages.

### 3.3. The Efficacy of Phage Cocktails In Vivo

#### 3.3.1. Lytic Activity of Single Phage or Cocktails Using the PKA Assay

**Figure 5:**
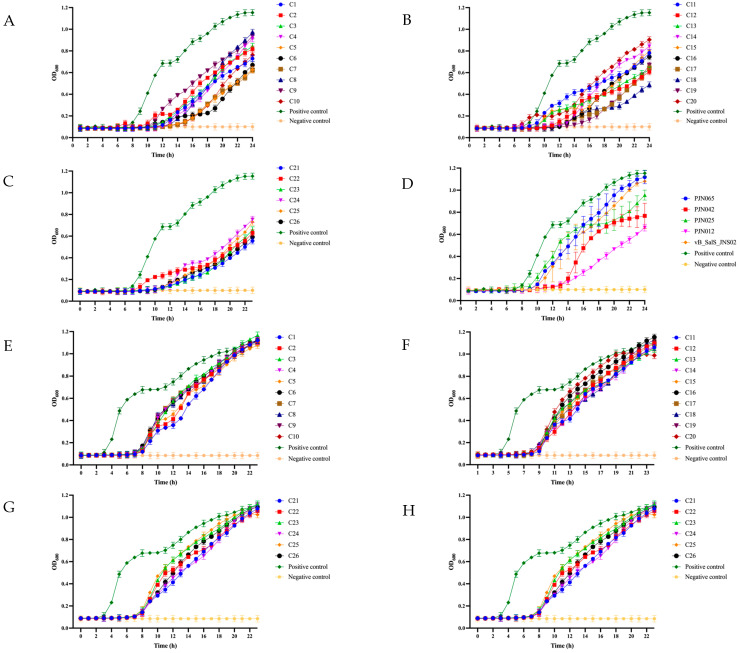
Lytic activity of 5 individual and 26 phage cocktails against two *Salmonella* strains at 37 °C. (**A**) Two phages for 015. (**B**) Three phages for 015. (**C**) Four and five phages for 015. (**D**) Individual phages for 015. (**E**) Two phages for 024. (**F**) Three phages for 024. (**G**) Four and five phages for 024. (**H**) Individual phages for 024.

After 24 h of incubation, all 26 phage cocktail combinations and each individual phage significantly reduced the OD_600_ values of *S*. *enteritidis* 024 and *S. typhimurium* 015, with statistical significance (*p* < 0.0001), when compared to the OD_600_ value of the positive control group. For the 015 strain, after 24 h of incubation, the OD_600_ values of different phage combinations were as follows: among two-phage combinations (C1–C10), C7 had the lowest OD_600_ value (0.6805); among three-phage combinations (C11–C20), C18 showed the lowest value (0.5415); among four-phage combinations (C21–C25), C21 had the lowest reading (0.608); and the five-phage combination C26 had an OD_600_ value of 0.6435 at this time (Figure 5A–D). Further analysis demonstrated that all four combinations (C7, C18, C21, C26) exhibited significantly better bacteriostatic effects than individual phages: the *p*-value for C26 vs. the individual phage PJN012 was 0.043, while *p*-values for the other combinations vs. individual phages were all <0.0001, indicating these phage cocktail combinations had more significant bacteriostatic effects than individual phages (Supplementary Table S6).

For the strain 024, among the two-phage combinations (C1–C10), the C9 combination had the lowest OD_600_ value (1.0625), and its inhibitory effect was significantly stronger than that of the four individual phages (JNS02, PJN012, PJN025, PJN042)—with *p*-values < 0.0001 for JNS02, PJN012, and PJN025, and *p* = 0.0003 for PJN042 (Figure 5E, Supplementary Table S7). In the three-phage combinations (C11–C20), the C20 combination showed the lowest OD_600_ value (1.012) and outperformed all five individual phages significantly: *p* = 0.0026 when compared to JNS02, and *p* < 0.0001 for the other four phages (Figure 5F, Supplementary Table S7). For the four-phage combinations (C21–C25), the C25 combination had the lowest OD_600_ value (1.101) and was significantly more effective than four individual phages, with *p* = 0.0078 versus PJN042 and *p* < 0.0001 versus JNS02, PJN012, and PJN025. In contrast, the five-phage combination C26 only exhibited a significant advantage over JNS02 (*p* < 0.0001), with no statistically significant difference in effect relative to the other individual phages (Figure 5G, Supplementary Table S7).

#### 3.3.2. Use of Virulence Index Score Indicates the Presence of Synergy

When the virulence index of a phage cocktail is compared with that of a single phage, an increase in the virulence index for the cocktail suggests the presence of a synergistic effect. Conversely, a lower virulence index for the phage cocktail than for a single phage indicates the potential existence of antagonism. The results revealed that, among the 26 cocktails tested for strain 015, 18 cocktails displayed synergistic effects, whereas for strain 024, six cocktails demonstrated synergistic effects (Table 2). Furthermore, an examination of the data in Table 2 reveals a significant variation in the virulence index of the same phage or phage cocktail when targeting the two strains. Specifically, for strain 015, 17 out of the 26 cocktails exhibited virulence indices exceeding 0.5, suggesting a high level of virulence against this strain.

#### 3.3.3. Efficacy of Multiple Phage Cocktails

The cocktail efficiently inhibited the growth of the bacteria in vitro, and their lytic efficiency was tested in vivo to determine whether the lytic efficiency of the cocktail was consistent in vitro and in vivo. For strain 015, the phage cocktails tested were C7 (two phages), C18 (three phages), C21 (four phages), and C26 (five phages), and for strain 024, the phage cocktails tested were C9 (two phages), C20 (three phages), C25 (four phages), and C26 (five phages).

**Figure 6:**
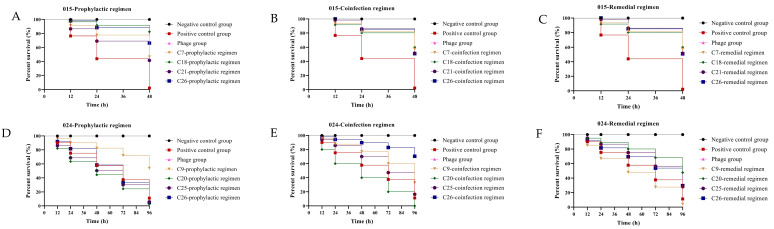
Four phage cocktails with excellent in vitro bacteriostasis against corresponding bacteria-infected *G. mellonella*: *Salmonella* strain 015 (**A**–**C**) and 024 (**D**–**F**).

During the experiment, no mortality was observed in the larvae of the negative control group and all phage-only treatment groups (hereafter referred to as “phage groups”). Given the large number of phage groups and their consistent survival status, all phage groups were collectively represented as “phage group” in Figure 6 to simplify data presentation. In contrast, in the positive control group, the survival rate of larvae infected with *Salmonella* strain 015 was only 5% at 48 h, while that of larvae infected with strain 024 was 30% at 96 h, indicating a significant difference in the lethality of the two strains to the larvae.

The phage cocktails exhibited varying effects on improving larval survival rates under different application modes. When the cocktails were used prophylactically, for strain 015, the survival rates of larvae treated with the four phage cocktails (C7, C18, C21, and C26) were all significantly higher than that of the positive control group (*p* < 0.0001), with the C18 group showing the best efficacy (survival rate up to 90%); for strain 024, however, only the C9 phage cocktail group achieved a significantly higher survival rate than the positive control group, with the survival rate reaching 75% (*p* < 0.0001). When *G. mellonella* infected with *Salmonella* were treated with co-infection phage therapy, for strain 015, the survival rates of the C7, C18, C21, and C26 groups remained significantly higher than that of the positive control group (*p* < 0.0001), and the C18 group still performed optimally (survival rate of 75%); for strain 024, the C26 group demonstrated the best effect, with the survival rate increased to 85% (*p* < 0.0001). In the remedial mode, for strain 015, the remedial group yielded results consistent with the co-infection group, and within these groups, the C18 group exhibited the most favorable outcome, with a survival rate reaching as high as 75%; for strain 024, the C20 group stood out with the highest survival rate of up to 70% (*p* < 0.0001).

### 3.4. Biofilm Eradication

#### 3.4.1. Bacteriophage-Mediated Inhibition of Biofilm Formation

##### *S*. Enteritidis 23C04 Strain

In the study on the 23C04 bacterial strain, individual bacteriophages showed varying inhibitory effects on biofilm growth compared to the positive control group (Supplementary Figure S2W). At 24 h, PJN012 and PJN065 (*p* < 0.05) significantly inhibited biofilm growth, as did PJN042 with a stronger effect (*p* < 0.01); their respective inhibition rates were 43.1%, 90.72%, and 69.95%. Notably, PJN025 maintained continuous and significant inhibitory activity at both 24 and 48 h (*p* < 0.05), with inhibition rates of 50.04% and 21.36% respectively.

**Figure 7:**
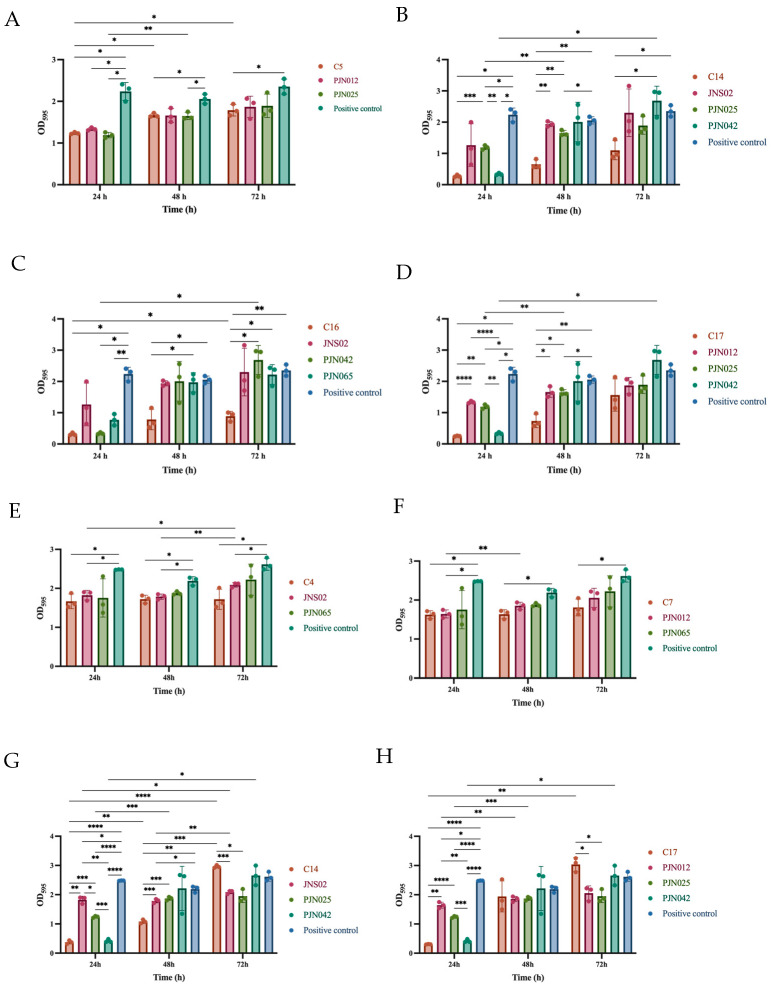
Effects of 5 individual phages and 4 phage cocktails on biofilms of two *Salmonella* strains at different incubation times (24 h, 48 h, 72 h). (**A**–**D**) *S*. Enteritidis strain 23C04. (**E**–**H**) *S*. Typhimurium strain 1804005. Each point in the figure represents an individual biological replicate. The overall data (e.g., group means) are the average of three independent replicates, and the error bars indicate the standard deviation (SD) calculated from these replicates, reflecting data variability. Statistical analysis was performed using two-way ANOVA with Tukey’s multiple comparisons test to compare all conditions. Significance levels are indicated as: * *p* ≤ 0.05; ** *p* ≤ 0.01; *** *p* ≤ 0.001; **** *p* ≤ 0.0001.

For the 26 tested bacteriophage cocktail combinations, their inhibitory capacities varied with incubation time compared to the positive control (Figure 7A–D, Supplementary Figure S2): At 24 h, 23 combinations (C1–3, C5–12, C14–21, and C23–26) significantly suppressed biofilm growth (*p* < 0.05), with C17 achieving the highest inhibition rate (95%); and the OD_595_ value of C17 was significantly lower than that of PJN012 and PJN025 (*p* < 0.01, Figure 7D). At 48 h, 6 combinations (C2, C5, C9, C14, C16, and C17) still retained significant inhibitory activity (*p* < 0.05), and C14 led with an inhibition rate of 73.46%; furthermore, the OD_595_ value of C14 was significantly lower than that of JNS02 and PJN025 (*p* < 0.01, Figure 7B). Even at 72 h of incubation, 5 combinations (C5, C10, C14, C16, and C26) remained effective (*p* < 0.05), with C16 showing the highest inhibition rate (66.72%); and the OD_595_ value of C16 was significantly lower than that of PJN042 and PJN065 (*p* < 0.05, Figure 7C). Three combinations (C5, C14, and C16) consistently and significantly inhibited biofilm growth at 24, 48, and 72 h, with respective inhibition rates as follows: C5 (47.78%, 20.42%, 25.78%), C14 (93.74%, 73.46%, 57.04%), and C16 (91.83%, 66.65%, 66.72%).

In conclusion, for rapid, potent, and stable inhibition of 23C04 strain biofilm growth, the C17 combination (at 24 h) is optimal. If the long-term stability of the inhibition rate (24–72 h) is required, C14 and C16 serve as reliable alternatives.

##### *S*. Typhimurium Strain 1804005

In the study on the 1804005 bacterial strain, individual bacteriophages displayed distinct differences in inhibiting biofilm growth (Supplementary Figure S3W). Compared to the positive control group, PJN012 significantly suppressed biofilm growth only at 24 h of incubation (*p* < 0.05), with an inhibition rate of 36.25%. PJN025 exhibited time-specific activity: it significantly inhibited biofilm growth at both 24 h (*p* < 0.0001) and 72 h (*p* < 0.05), with the 24 h effect being particularly prominent (inhibition rates: 53.71% and 27.19%, respectively) (Figure 7A). At 24 h, PJN042 showed extremely strong inhibitory capacity, significantly reducing biofilm growth (*p* < 0.0001) with an inhibition rate as high as 89.33%. Notably, JNS02 demonstrated excellent sustained activity, significantly inhibiting biofilm growth at three time points (24, 48, and 72 h; *p* < 0.05) with inhibition rates of 28.44%, 19.59%, and 21.45%, respectively.

For the 26 bacteriophage cocktail combinations (vs. the positive control group), 20 combinations (C1–12, C14–18, C20–22) significantly inhibited biofilm growth at 24 h (*p* < 0.05), with C17 achieving the highest inhibition rate (94.21%), and the OD_595_ value of C17 was significantly lower than that of PJN012 and PJN025 (*p* < 0.01) (Figure 7H). By 48 h, only 5 combinations (C2, C4, C7, C14, C26) retained significant inhibitory activity (*p* < 0.05), and C14 performed exceptionally well (*p* < 0.01) with an inhibition rate of 45.36%; furthermore, the OD_595_ value of C14 was significantly lower than that of JNS02 and PJN025 (*p* < 0.001) (Figure 7G). At 72 h, 7 combinations (C1, C4, C5, C7, C9, C13, C19) still significantly suppressed biofilm growth (*p* < 0.05), with C4 showing the highest inhibition rate (36.54%). Two phage cocktails C4 and C7 could consistently and significantly inhibit the biofilm growth at 24, 48, and 72 h, with respective inhibition rates as follows: C4 (35.29%, 22.66%, 36.54%) and C7 (37.05%, 26.84%, 32.83%).

In summary, for rapid inhibition of 1804005 strain biofilm growth, the C17 combination is recommended, as it exhibits optimal activity at the 24 h incubation time point.

#### 3.4.2. Bacteriophage-Mediated Removal of 24 H Formed Biofilms

This study systematically assessed the biofilm removal efficacy of individual bacteriophages and 26 bacteriophage cocktail combinations against 24 h pre-grown biofilms, evaluating their performance across six time points: 4, 6, 8, 12, 24, and 48 h post-treatment.

##### *S*. Enteritidis 23C04 Strain

The biofilm removal efficacy of individual bacteriophages against strain 23C04 exhibited a distinct time-dependent pattern (Supplementary Figure S4X). In comparison to the positive control group, only two bacteriophages, PJN012 and PJN042, achieved significant biofilm removal after 6 h of treatment (*p* < 0.05), with removal rates of 59.65% and 31.31%, respectively. When the treatment duration was extended to 12 h, the number of bacteriophages with significant removal effects increased to four strains (JNS02, PJN012, PJN025, and PJN065), and the statistical significance became more pronounced (*p* < 0.01); their respective reduction rates were 62.83%, 67.57%, 73.86%, and 66.82%. Even after 48 h of action, four bacteriophages (JNS02, PJN065, PJN025, and PJN012) retained significant removal activity. Among these, JNS02 and PJN065 showed significance at *p* < 0.05, while PJN025 (*p* < 0.01) and PJN012 (*p* < 0.001) demonstrated superior efficacy, with removal rates of 40.36%, 36.57%, 40.43%, and 32.85%, respectively.

**Figure 8:**
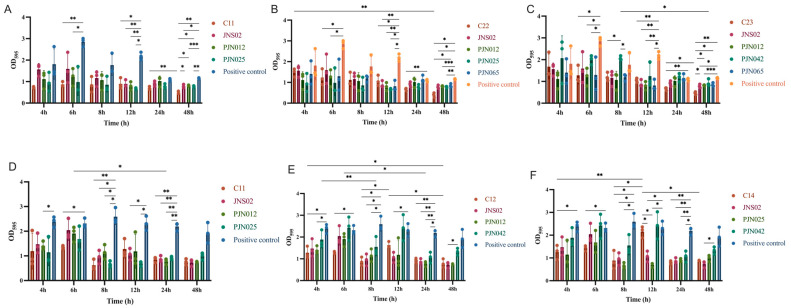
Impact of 5 single phage and 3 phage cocktails on biofilm removal of two *Salmonella* strains across diverse incubation durations: 4 h, 8 h, 12 h, 24 h, and 48 h. (**A**–**C**) *S*. Enteritidis strain 23C04. (**D**–**F**) *S*. Typhimurium strain 1804005. Each point in the figure represents an individual biological replicate. The overall data (e.g., group means) are the average of three independent replicates, and the error bars indicate the standard deviation (SD) calculated from these replicates, reflecting data variability. Statistical analysis was performed using two-way ANOVA with Tukey’s multiple comparisons test to compare all conditions. Significance levels are indicated as: * *p* ≤ 0.05; ** *p* ≤ 0.01; *** *p* ≤ 0.001.

The biofilm removal performance of the 26 cocktails also varied with treatment time, and their overall effectiveness—particularly in terms of long-term sustainability—outperformed that of individual bacteriophages (Figure 8A–C, Supplementary Figure S4). Compared to the positive control group, none of the cocktails exhibited significant removal activity during short-term treatment (4 and 8 h) (*p* > 0.05), suggesting that these combinations require a certain period to exert their effects. After 6 h of treatment, a total of 19 cocktails (C1, C3–8, C11–18, C22–24, and C26) achieved a significant reduction in biofilm (*p* < 0.05). Among them, the C11 had the highest biofilm reduction rate, reaching up to 72.53%, but its removal effect showed no significant difference compared with that of a single phage. In addition, 5 phage combinations (C12, C14, C17, C24, and C26) exhibited better removal effects than a single phage, with the C24 combination standing out, achieving a reduction rate of 69.69%. At the 12 h time point, the number of cocktails that achieved a significant biofilm reduction increased to 22 (C1–4, C6, C9–14, and C16–26, *p* < 0.05). The C11 again ranked first with a reduction rate of 62.8%, yet its biofilm removal capacity was inferior to that of individual bacteriophages (JNS02, PJN012, PJN025, and PJN065). After 24 h of treatment, 12 cocktails (C4, C11–13, C16–19, and C21–24) still maintained significant biofilm removal activity (*p* < 0.05). However, none of these 12 cocktails showed a significant difference in removal effect compared with a single phage. Among them, the C23 performed relatively prominently, with a maximum reduction rate of 53.27%. By 48 h, a total of 24 cocktails (C1–9 and C11–25) exhibited significant biofilm removal effects (*p* < 0.05), and 18 of these cocktails had significantly better removal effects than a single phage—making this time point the stage with the “highest proportion of high-efficiency combinations” in the experiment. Notably, at this stage, the C22 had the highest reduction rate, an impressive 82.78%, and its OD_595_ value was significantly lower than that of PJN065 (*p* < 0.05), which further confirms its excellent removal efficacy.

If the requirement is “short-term rapid onset of efficacy plus full-cycle stability”, the C24 combination is recommended. It achieves a biofilm removal rate of 69.69% at 6 h and maintains a rate of 78.7% at 48 h, with its efficacy being significantly superior to that of individual bacteriophages.

##### *S*. Typhimurium Strain 1804005

The biofilm removal efficacy of bacteriophages against strain 1804005 differs significantly from that against strain 23C04. When examining the performance of individual bacteriophages, comparisons with the positive control group revealed the following patterns (Supplementary Figure S5X): At 4 h post-treatment, only PJN012 and PJN065 achieved significant biofilm reduction (*p* < 0.05), with removal rates of 43.22% and 53.7%, respectively. By 8 h, the number of bacteriophages exhibiting significant removal activity increased to 3 strains (JNS02, PJN012, PJN025) (*p* < 0.05), with respective reduction rates of 65.88%, 57.86%, and 77.33%; among these, PJN025 demonstrated the highest removal efficiency. At the 12 h mark, only JNS02 and PJN025 maintained significant removal activity (*p* < 0.05), with reduction rates of 56.68% and 73.54%, respectively. The number of bacteriophages capable of significant biofilm removal peaked at 24 h, totaling 5 strains (JNS02, PJN012, PJN025, PJN042, PJN065) (*p* < 0.05), and their removal rates were 65.79%, 71.24%, 64.37%, 56.34%, and 60.30% in sequence.

Regarding the biofilm removal efficacy of bacteriophage cocktails, the specific findings relative to the positive control group (Figure 8 D–F, Supplementary Figure S5) were summarized as follows: At 4 h, 8 cocktails (C12, C14, C17, C21–24, C26) achieved significant biofilm removal (*p* < 0.05). Among these, the C22 exhibited standout performance with the highest removal rate (57.73%); however, its removal efficacy did not differ significantly from that of individual bacteriophages (Supplementary Figure S5S). Among these 8 cocktails, only the OD_595_ value of the C14 was significantly lower than those of JNS02 and PJN025, with a biofilm reduction rate of up to 49.85%. However, this removal rate was lower than that of PJN065 at the same time point (53.7%).

By 6 h, the number of cocktails with significant removal efficacy decreased to 6 (C4, C11, C12, C14, C18, C21) (*p* < 0.05), with the C18 performing optimally at a reduction rate of 50.89%. Notably, none of these 6 cocktails demonstrated significantly higher removal efficacy than individual bacteriophages (*p* > 0.05).

At 8 h, the count of cocktails with significant removal efficacy rebounded to 8 (C1, C2, C5, C11, C12, C14, C17, C24) (*p* < 0.05). C11 showed excellent removal efficiency, reaching a rate of 79.35%, yet its efficacy remained statistically no comparable to that of individual bacteriophages (*p* > 0.05). In contrast, two cocktails—C2 and C5—exhibited significant differences in removal efficacy relative to individual bacteriophages, with respective removal rates of 46.45% and 49.17%. Importantly, both rates were lower than the removal rates of the three individual bacteriophages (JNS02, PJN012, and PJN025) at the same time point.

At 12 h, only C19 resulted in a biofilm level significantly lower than that of the positive control group (*p* < 0.05), with a reduction rate of 52.67%. Nevertheless, this removal efficacy was weaker than that of the individual bacteriophages JNS02 and PJN025 (56.68% and 73.54%, respectively). By 24 h, the number of cocktails with significant removal efficacy peaked at 25 (C1–9, C11–26) (*p* < 0.05). However, none of these 25 combinations showed a significant difference in removal efficacy compared to individual bacteriophages (*p* > 0.05). The C19 performed best at this time point, with a reduction rate of 72.04%, while the individual bacteriophage PJN012 also achieved a removal rate of 71.24% during the same period. At 48 h, compared with the positive control group, the bacteriophage cocktail combinations showed no significant difference in biofilm removal efficacy.

## 4. Discussion

Given the profound impact of *Salmonella* on public health and the widespread emergence of multidrug-resistant strains, bacteriophages have garnered significant attention as promising alternatives for the control of *Salmonella* infection in recent years. In this study, three newly isolated bacteriophages were characterized in detail. Using these three novel phages alongside two previously isolated phages, various combinations of bacteriophages were formulated. The in vitro and in vivo efficacy of these combinations was then assessed.

An important criterion for phage composition is the host range of the individual phages, ideally with partially overlapping and supplementary host ranges. It is well known that phage cocktails can expand the lytic spectrum, and in this study, the cocktail combination of five phages significantly expanded the lytic spectrum of eight *Salmonella* serotypes as well as *E. coli*. The PKA approach serves as a pivotal tool in evaluating the efficacy of phages in the liquid environment, facilitating the assembly of phage cocktails. By selecting phages from a phage pool on the basis of their single killing assay curves, this method involves monitoring the optical density of exponentially growing bacteria infected with a specific phage concentration over time. Phages demonstrating robust lytic activity, evident from reduced bacterial optical density and distinct lytic profiles, are then chosen to formulate potent phage cocktails.

### 4.1. Analysis of Bacteriophage Characteristics

The research on the thermostability of bacteriophages is centered on their practical application needs. Bacteriophages with a certain degree of heat stability can not only effectively extend their own storage period but also ensure the maintenance of a relatively high titer during storage and application. Among the three strains of bacteriophages involved in this study, PJN042 can survive in an environment of 30–60 °C; in contrast, PJN012 and PJN065 exhibit better thermostability, as they can still maintain a relatively high titer within the temperature range of 30–70 °C, which provides conditions for their wider application scenarios. The pH stability of bacteriophages directly influences the selection of animal administration routes, and this influence is particularly prominent in the context of oral administration. This is because after oral ingestion, bacteriophages traverse the entire length of the animal’s digestive tract, during which they inevitably come into contact with the acidic environment within the tract. Consequently, their pH tolerance capacity becomes a critical prerequisite for the feasibility of oral administration. The three strains of bacteriophages involved in this study can maintain high activity across the broad pH range of 2 to 12. This characteristic provides important evidence for the adoption of oral administration for these bacteriophages, suggesting that this administration route holds potential for practical application.

To date, no optimal latent periods or burst sizes have been established for bacteriophages in therapeutic and prophylactic applications. However, bacteriophages characterized by rapid replication and large burst sizes are typically favored [36]. The latent periods of the three bacteriophages in this study range from 60 to 70 min, among which PJN065 exhibits a relatively higher burst size. Environmental factors such as temperature, pH, culture medium, cation availability, host organism, host density, and host physiological status all exhibit variations that can affect the latent period and burst size of different phages. These variations, in turn, render it difficult to compare these phage characteristics with those observed under in vivo conditions [37,38].

The lytic efficacy of phages is closely related to the MOI. A low MOI may be insufficient for effectively addressing infection, whereas an excessively high MOI can expedite bacterial resistance [39]. Given the potential sparsity of contact between phages and their target bacteria within a biological context, an MOI of at least 10 is generally advised to guarantee that an impressive 99.9% of host bacterial cells are infected by at least one phage particle [40]. Numerous studies have explored the impact of varying MOIs on the lytic capabilities of individual phages or phage cocktails. However, our research specifically concentrated on assessing the lytic performance of phage cocktails at an MOI of 10. Our primary interest lies in examining the variations in lytic efficacy among different phage combinations under a constant MOI, which enables meaningful comparisons and insights.

### 4.2. Differences Between In Vitro and In Vivo Efficacies of Phage Cocktails

Based on the results of PKA, the phage cocktails exhibited greater lytic efficacy against *S. Enteritidis* 015 than against *S. Typhimurium* 024, with several cocktails consistently inhibiting the growth of *S. Enteritidis* 015 for up to 13 h. Although all phage combinations exhibited lytic performance superior to that of single phages in our study, notably, a previously reported phage cocktail was capable of inhibiting bacterial growth for 20 h [20]. Our findings align with those of another study [41].

Given their innate immune response, which mirrors that of larger vertebrates [42,43], *G. mellonella* larvae have emerged as a successful model for assessing the efficacy of phage lysis in vivo. This infection model boasts simplicity, cost-effectiveness, and a less intricate ethical framework than mammalian studies do [44], enabling the examination of a broader spectrum of formulations, dosages, and treatment protocols. After selecting the optimal phage cocktails in vitro, we evaluated the performance of the two-, three-, four-, and five-phage cocktails in vivo. Results from animal experiments using *G. mellonella* demonstrated that, while all tested phage cocktail combinations exerted significant bacteriostatic effects in vitro, some combinations showed no corresponding bacteriostatic activity whatsoever when tested in vivo. This highlights a discrepancy between the findings of the in vivo and in vitro experiments. In terms of the mode of phage application, our results were consistent with the previous studies [25,45], which demonstrated prophylactic or coinfection regimens are preferable to remedial regimens.

A core challenge in phage therapy research is the discrepancy between phage cocktails in vitro and in vivo efficacies, rooted in simplified in vitro systems versus complex in vivo physiological microenvironments—with four key mechanisms explaining this: host innate immunity (e.g., in *G. mellonella*) disrupts stable in vitro phage–bacterium interactions by clearing phages and isolating bacteria; physical barriers (e.g., tissues) block phage access to infection sites and chemical fluctuations (e.g., pH/ion levels) inhibit phage activity, unlike homogeneous in vitro systems ensuring full phage–bacterium contact; in vivo, phage synergy shifts to competition and bacteria develop new resistance, rendering in vitro-identified synergistic combinations ineffective; and efficacy metrics differ—in vitro focuses on “reduced bacterial counts,” while in vivo requires “decreased virulence” and “inhibited colonization,” causing “in vitro efficacy but in vivo ineffectiveness” for phages that only suppress in vitro bacterial growth.

### 4.3. The Inhibitory and Removal Effects of Bacteriophages on Biofilms

*Salmonella* can form biofilms, which are intricate aggregations of bacteria that adhere to surfaces and secrete protective extracellular polymeric substances, significantly hindering the efficacy of antibiotics. In a previous study, researchers employed phages of varying titer to assess the biofilm reduction rate under identical conditions and ultimately discovered that a titer of 10^9^ PFU/mL resulted in the most effective eradication [21]. Consequently, we used 10^9^ PFU/mL for our experiment. The results of this study showed that in terms of inhibiting biofilm growth, phage cocktails exhibited significant advantages compared to single phages. Among them, the C17 combination had the most prominent inhibitory effect, as it achieved the strongest inhibition on the biofilm growth of two tested bacterial strains after 24 h of action. However, when it came to eliminating pre-formed biofilms (pre-cultured for 24 h), phage cocktails showed distinct differences in their effects on the two bacterial strains. For *S*. Enteritidis 23C04, phage cocktails demonstrated excellent elimination ability after 48 h of action, and the biofilm elimination rates of all 18 cocktail combinations were significantly better than those of single phages. In contrast, for *S*. Typhimurium 1804005, the elimination effect of phage cocktails was very limited, and no advantage was observed compared to single phages. So we hypothesize that the phage cocktail in this study has an inhibitory effect rather than a biocidal effect, which is consistent with previous results for the phage cocktail UPWr_S134 [21].

The observation that the phage cocktail exerts an inhibitory rather than a biocidal effect can be attributed to four key factors: (1) the complex, multilayered structure of mature biofilms physically blocks phage penetration, lowering efficacy against established biofilms; (2) phages infect and lyse exponentially growing bacteria more efficiently than stationary-phase ones, with bacteria during early biofilm formation—when they actively attach and multiply—being more susceptible to phage attack; (3) a preventive strategy (phage application during bacterial attachment) disrupts initial colonization more effectively than eradicating already formed biofilms; (4) mature biofilms need longer phage exposure due to their resistance mechanisms, whereas early attachment-phase intervention minimizes the time required for effective control.

### 4.4. The Antagonistic or Synergistic Interactions Among Phages

Over time, researchers have investigated the existence of antagonistic or synergistic interactions among phages within a phage cocktail. The concept of the virulence index offers some insight into these interactions. Building upon the viewpoint employed by Haines et al. [46], we also observed instances of both antagonism and synergy among phages through the application of the virulence index. Notably, despite these antagonistic or synergistic interactions, all combinations of phages demonstrated a propensity to surpass individual phages in terms of lytic performance. Although PJN012, PJN065, and JNS02 belong to the *Jerseyvirus* genus, synergistic effects are evident not only in two-phage combinations among these three phages but also in combinations with the other two phages, PJN025 and PJN042, involving three-phage, four-phage, or even five-phage cocktails. Although the mechanism underlying this synergistic effect remains unclear, JNS02 is present in more than half of the synergistic combinations. These findings suggest that JNS02 may play a pivotal role in enhancing the synergy among bacteriophages in the cocktail. In addition to phage genera and their combinations, factors such as target bacterial strains and incubation times can also influence phage-phage interactions, as evidenced in other studies [47].

## 5. Conclusions

In conclusion, our study revealed that the efficacy of phage cocktails was influenced not only by biological factors such as phage type and targeted bacterial strain but also by the intended application and duration of exposure. Notably, the lysis properties of phages may differ between in vitro and in vivo environments. Hence, to develop highly effective phage cocktails, it is imperative to comprehensively consider and evaluate these biological factors and their influences, ensuring optimal synergy among the phages.

## Figures and Tables

**Table 1:** List of phage cocktails tested in this study.

Phage Cocktails	Phages Included Within the Cocktail	Number of Cocktails
Two-phage cocktails	JNS02 + PJN012	C1
	JNS02 + PJN025	C2
	JNS02 + PJN042	C3
	JNS02 + PJN065	C4
	PJN012 + PJN025	C5
	PJN012 + PJN042	C6
	PJN012 + PJN065	C7
	PJN025 + PJN042	C8
	PJN025 + PJN065	C9
	PJN042 + PJN065	C10
Three-phage cocktails	JNS02 + PJN012 + PJN025	C11
	JNS02 + PJN012 + PJN042	C12
	JNS02 + PJN012 + PJN065	C13
	JNS02 + PJN025 + PJN042	C14
	JNS02 + PJN025 + PJN065	C15
	JNS02 + PJN042 + PJN065	C16
	PJN012 + PJN025 + PJN042	C17
	PJN012 + PJN042 + PJN065	C18
	PJN012 + PJN025 + PJN065	C19
	PJN025 + PJN042 + PJN065	C20
Four-phage cocktails	JNS02 + PJN012 + PJN025 + PJN042	C21
	JNS02 + PJN012 + PJN025 + PJN065	C22
	JNS02 + PJN012 + PJN042 + PJN065	C23
	JNS02 + PJN025 + PJN042 + PJN065	C24
	PJN012 + PJN025 + PJN042 + PJN065	C25
Five-phage cocktails	JNS02 + PJN012 + PJN025 + PJN042 + PJN065	C26

**Table 2:** The virulence index of individual phage and phage cocktails across two strains.

Phage/Phage Cocktail	*S*. Enteritis Strain 015	*S*. Typhimurium Strain 024
PJN012	0.5921	0.3094
PJN025	0.2603	0.3094
PJN042	0.4456	0.2755
PJN065	0.2011	0.2773
JNS02	0.2441	0.1471
C1	0.5243	0.3222
C2	0.4385	0.2860
C3	0.4709	0.2271
C4	0.4975	0.2452
C5	0.6352	0.2966
C6	0.6252	0.2423
C7	0.6367	0.2510
C8	0.4633	0.2434
C9	0.3703	0.2434
C10	0.5911	0.3222
C11	0.4171	0.3152
C12	0.5705	0.286
C13	0.5316	0.2825
C14	0.4677	0.3141
C15	0.4923	0.2936
C16	0.5435	0.2125
C17	0.6307	0.2697
C18	0.6716	0.2808
C19	0.6467	0.2773
C20	0.3679	0.2061
C21	0.6482	0.3275
C22	0.5583	0.2942
C23	0.6194	0.2271
C24	0.5407	0.3199
C25	0.5773	0.2242
C26	0.6206	0.2744

## Data Availability

The authors declare that the data supporting the findings of this study are available within the paper and its Supplementary Information Files. Should any raw data files be needed in another format, they are available from the corresponding author upon reasonable request.

## Supplementary Materials

The following supporting information can be downloaded at: https://www.mdpi.com/article/10.3390/v17101363/s1.
