# Supplementary material for: Design of Lytic Phage Cocktails Targeting Salmonella: Synergistic Effects Based on In Vitro Lysis, In Vivo Protection, and Biofilm Intervention
Source: Viruses. 2025 Oct 12;17(10):1363. doi: 10.3390/v17101363 (PMC12568117; doi:10.3390/v17101363)
Supplement: Supplementary file 1 [file viruses-17-01363-s001.zip › Supplementary Figues.pdf]

Supplementary Figures

A

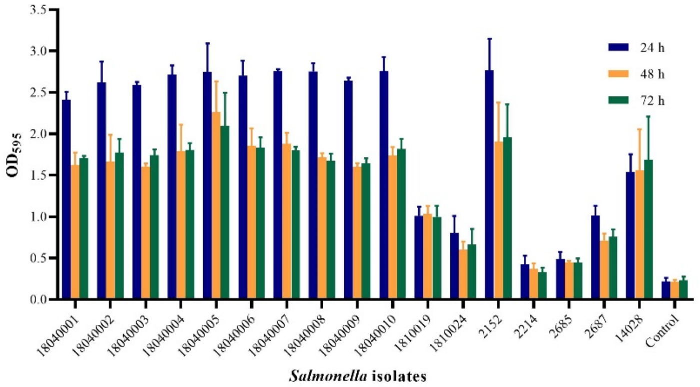

B

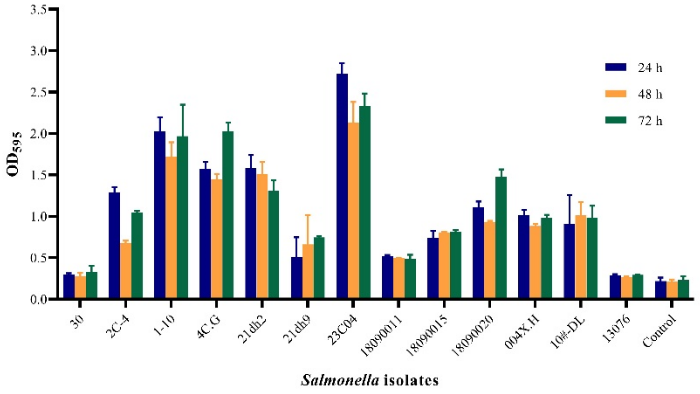

Figure S1. Determination of biofilm-forming ability of 30 *Salmonella* strains at different times. (A) *S*. Typhimurium strains. (B) *S*. Enteritidis strains.

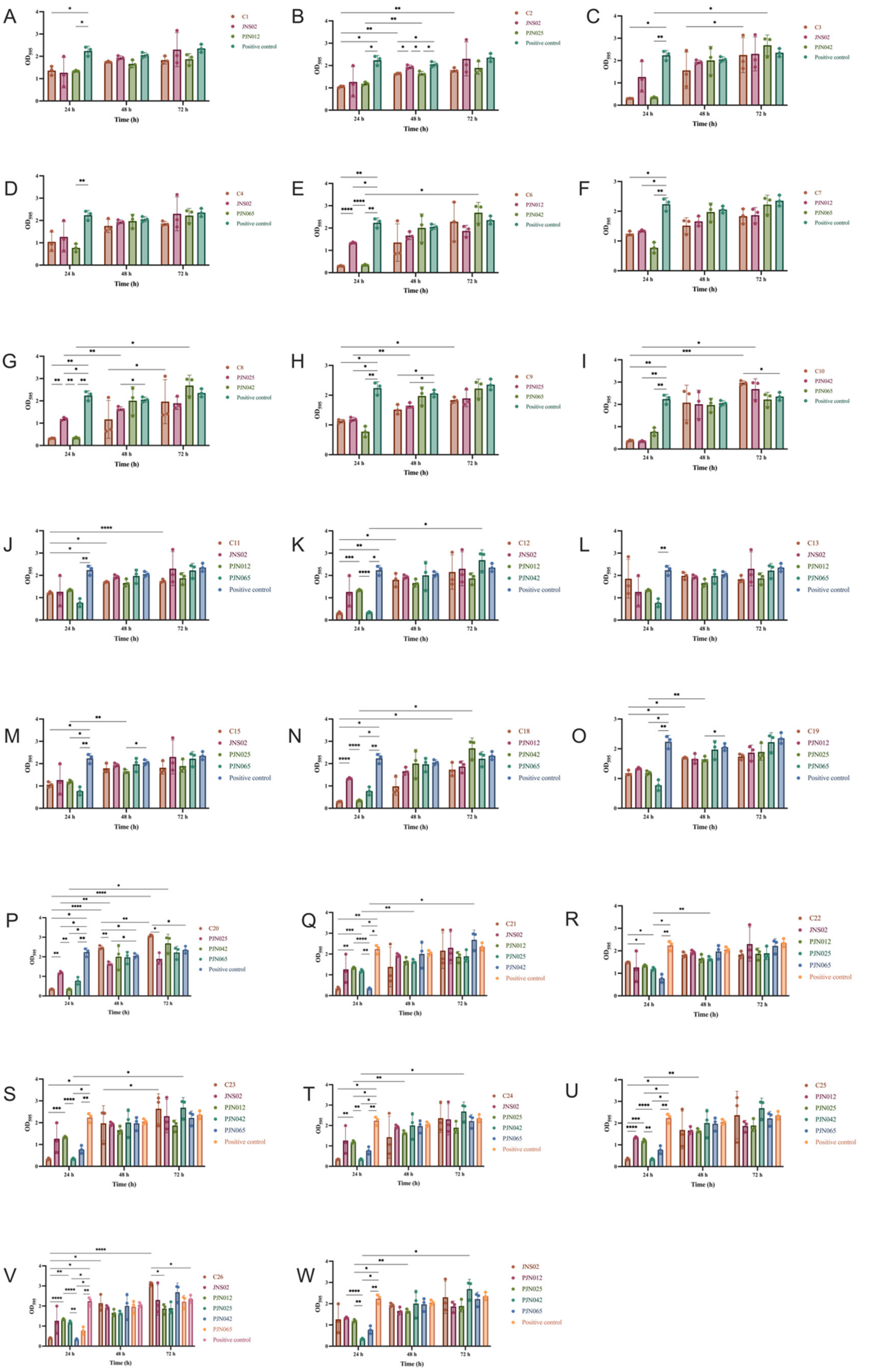

Figure S2. Effects of 5 individual phages and 22 phage cocktails on biofilms of S. Enteritidis strain 23C04 at different incubation times (24 h, 48 h, 72 h). Each point in the figure

represents an individual biological replicate. The overall data (e.g., group means) are the average of three independent replicates, and the error bars indicate the standard deviation (SD) calculated from these replicates, reflecting data variability. Statistical analysis was performed using two-way ANOVA with Tukey's multiple comparisons test to compare all conditions. Significance levels are indicated as: * $p \leq 0.05$; ** $p \leq 0.01$; *** $p \leq 0.001$; **** $p \leq 0.0001$.

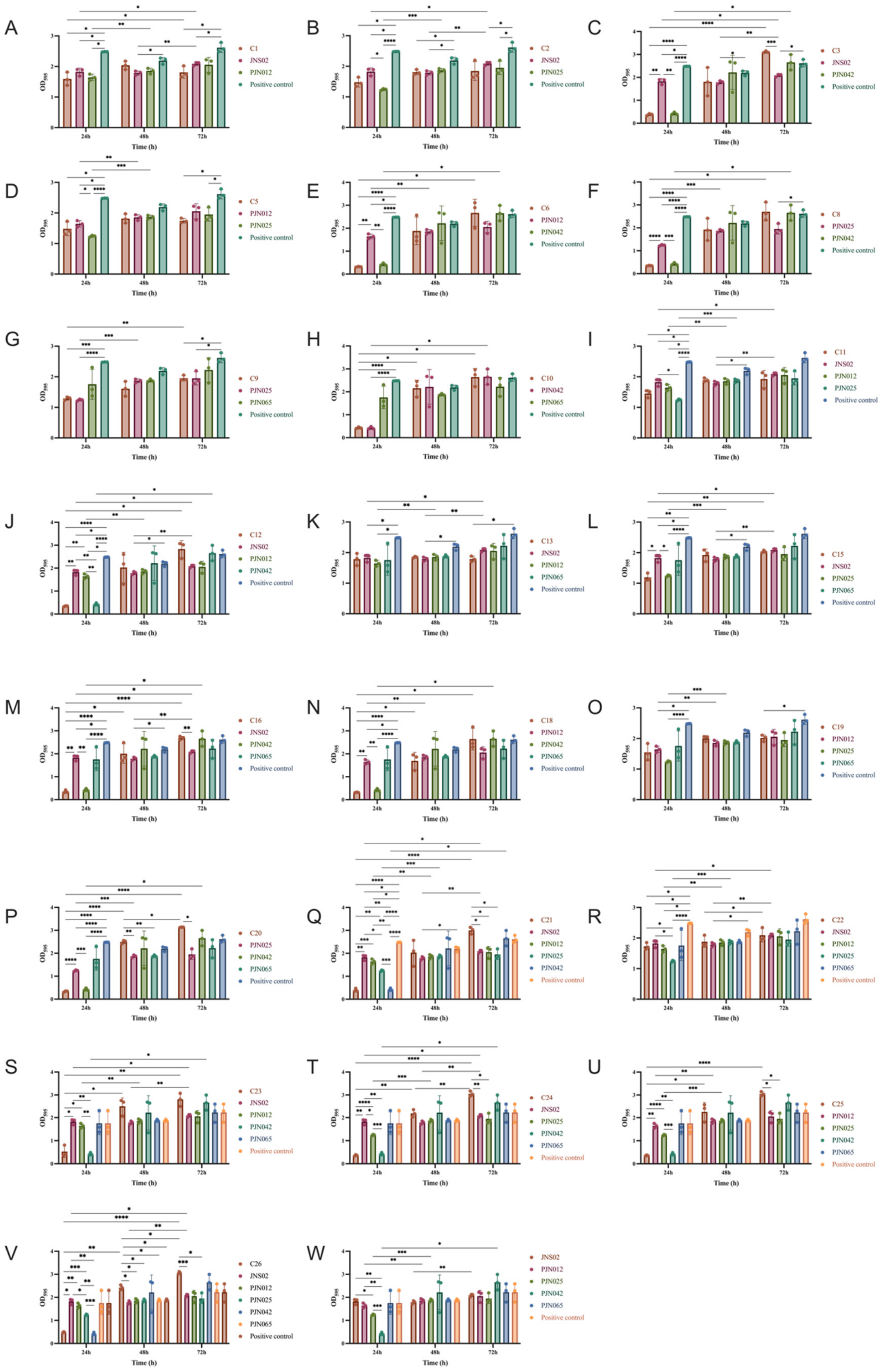

Figure S3. Effects of 5 individual phages and 22 phage cocktails on biofilms of S. Typhimurium strain 1804005 at different incubation times (24 h, 48 h, 72 h). Each point in

the figure represents an individual biological replicate. The overall data (e.g., group means) are the average of three independent replicates, and the error bars indicate the standard deviation (SD) calculated from these replicates, reflecting data variability. Statistical analysis was performed using two-way ANOVA with Tukey's multiple comparisons test to compare all conditions. Significance levels are indicated as: * $p \leq 0.05$; ** $p \leq 0.01$; *** $p \leq 0.001$; **** $p \leq 0.0001$.

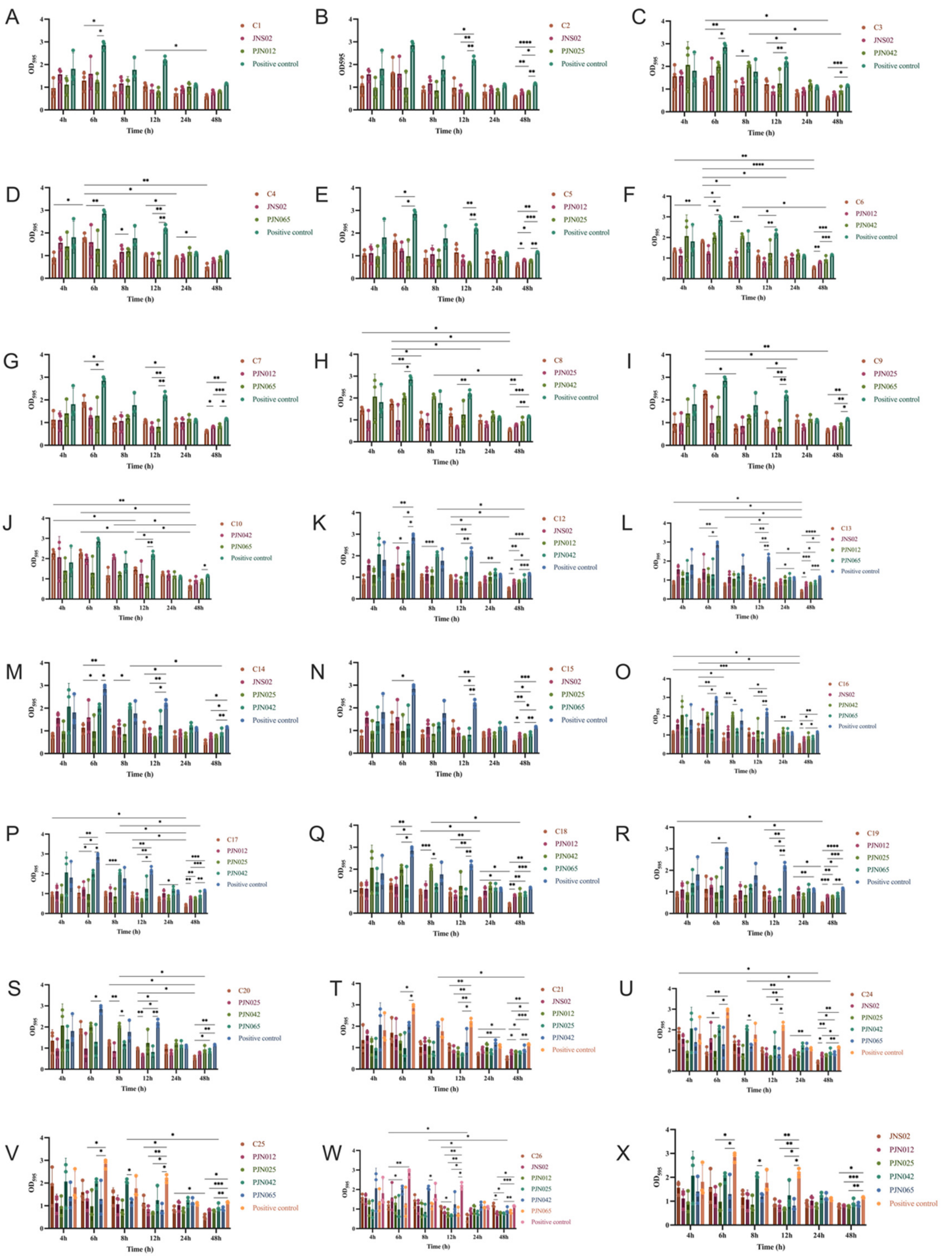

Figure S4. Impact of 5 single phage and 23 phage cocktails on biofilm removal of S. Enteritidis strain 23C04 strain across diverse incubation durations: 4 h, 8 h, 12 h, 24 h, and 48 h. Each point in the figure represents an individual biological replicate. The overall data (e.g., group means) are the average of three independent replicates, and the error bars indicate the standard deviation (SD) calculated from these replicates, reflecting data variability. Statistical analysis was performed using two-way ANOVA with Tukey's multiple comparisons test to compare all conditions. Significance levels are indicated as: * $p \leq 0.05$; ** $p \leq 0.01$; *** $p \leq 0.001$; **** $p \leq 0.0001$.

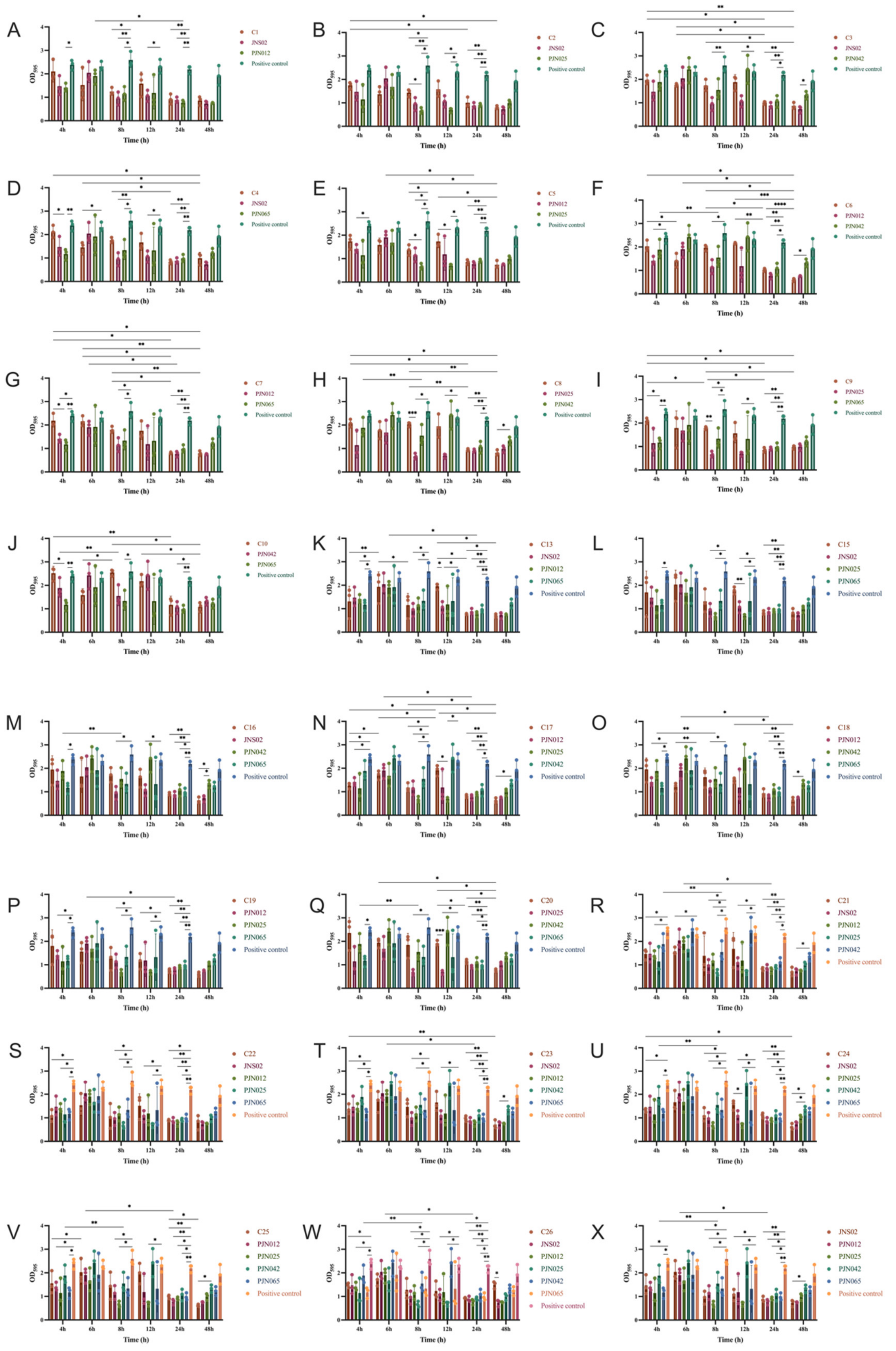

Figure S5. Impact of 5 single phage and 23 phage cocktails on biofilm removal of S. Typhimurium strain 1804005 across diverse incubation durations: 4 h, 8 h, 12 h, 24 h, and 48 h. Each point in the figure represents an individual biological replicate. The overall data

(e.g., group means) are the average of three independent replicates, and the error bars indicate the standard deviation (SD) calculated from these replicates, reflecting data variability. Statistical analysis was performed using two-way ANOVA with Tukey's multiple comparisons test to compare all conditions. Significance levels are indicated as: * $p \leq 0.05$; ** $p \leq 0.01$; *** $p \leq 0.001$; **** $p \leq 0.0001$.
