# Supplementary material for: Design of Lytic Phage Cocktails Targeting Salmonella: Synergistic Effects Based on In Vitro Lysis, In Vivo Protection, and Biofilm Intervention
Source: Viruses. 2025 Oct 12;17(10):1363. doi: 10.3390/v17101363 (PMC12568117; doi:10.3390/v17101363)
Supplement: Supplementary file 1 [file viruses-17-01363-s001.zip › Supplementary Tables.pdf]

Supplementary Table

Table S1. Information on the serotype and source of *Salmonella* strains used in the biofilm formation experiment

in-text number	Original ID	Animal source	Province	Serotype	Sampling Time
18040001	SSPZ18040001	pig	Zhejiang	*S*.Typhimurium	2019.07.08
18040002	SSPZ18040002	pig	Zhejiang	*S*.Typhimurium	2019.07.08
18040003	SSPZ18040003	pig	Zhejiang	*S*.Typhimurium	2019.07.08
18040004	SSPZ18040004	pig	Zhejiang	*S*.Typhimurium	2019.07.08
18040005	SSPZ18040005	pig	Zhejiang	*S*.Typhimurium	2019.07.08
18040006	SSPZ18040006	pig	Zhejiang	*S*.Typhimurium	2019.07.08
18040007	SSPZ18040007	pig	Zhejiang	*S*.Typhimurium	2019.07.08
18040008	SSPZ18040008	pig	Zhejiang	*S*.Typhimurium	2019.07.08
18040009	SSPZ18040009	pig	Zhejiang	*S*.Typhimurium	2019.07.08
18040010	SSPZ18040010	pig	Zhejiang	*S*.Typhimurium	2019.07.08
1810019	SSDZ1810019	duck	Zhejiang	*S*.Typhimurium	2019.07.08
1810024	SSDZ1810024	duck	Zhejiang	*S*.Typhimurium	2019.07.08
	SM-1826-ZD	Chick	Zhejiang	*S*.Typhimurium	2021.06.24
	SM-1827-ZD	Chick	Zhejiang	*S*.Typhimurium	2021.06.24
	SM-2005-ZD	Chick	Zhejiang	*S*.Typhimurium	2021.06.24
	SM-2008-ZD	Chick	Zhejiang	*S*.Typhimurium	2021.06.24
	SM-2010-ZD	Chick	Zhejiang	*S*.Typhimurium	2021.06.24
	SM-2073-ZD	Chick	Zhejiang	*S*.Typhimurium	2021.06.24
2152	SM-2152-ZD	Chick	Zhejiang	*S*.Typhimurium	2021.06.24
2214	SM-2214-ZD	Chick	Zhejiang	*S*.Typhimurium	2021.06.24
	SM-2239-ZD	Chick	Zhejiang	*S*.Typhimurium	2021.06.24
2685	SM-2685-ZD	Chick	Zhejiang	*S*.Typhimurium	2021.06.24
2687	SM-2687-ZD	Chick	Zhejiang	*S*.Typhimurium	2021.06.24
	SM-2304-ZD	Chick	Zhejiang	*S*.Typhimurium	2021.06.24
	SM-2648-ZD	Chick	Zhejiang	*S*.Typhimurium	2021.06.24
	SM-2679-ZD	Chick	Zhejiang	*S*.Typhimurium	2021.06.24
	SM-2685-ZD	Chick	Zhejiang	*S*.Typhimurium	2021.06.24
	SM-2686-ZD	Chick	Zhejiang	*S*.Typhimurium	2021.06.24
	SM-2687-ZD	Chick	Zhejiang	*S*.Typhimurium	2021.06.24
	SM-3292-ZD	Chick	Zhejiang	*S*.Typhimurium	2021.06.24
	SM-3863-ZD	Chick	Zhejiang	*S*.Typhimurium	2021.06.24
	SSDZ1806001	Pig	Zhejiang	*S*.Typhimurium	2019.07.08
	SSDZ18060093	Duck	Zhejiang	*S*.Typhimurium	2019.07.08
	SSDZ18060094	Duck	Zhejiang	*S*.Typhimurium	2019.07.08
	SSDZ18060101	Duck	Zhejiang	*S*.Typhimurium	2019.07.08
	SSDZ18060102	Duck	Zhejiang	*S*.Typhimurium	2019.07.08
	SSDZ18060104	Duck	Zhejiang	*S*.Typhimurium	2019.07.08
	SSDZ1810001	Duck	Zhejiang	*S*.Typhimurium	2019.07.08

	SSDZ1810002	Duck	Zhejiang	*S*.Typhimurium	2019.07.08
	SSDZ1810003	Duck	Zhejiang	*S*.Typhimurium	2019.07.08
	SSDZ1810004	Duck	Zhejiang	*S*.Typhimurium	2019.07.08
	SSDZ1810005	Duck	Zhejiang	*S*.Typhimurium	2019.07.08
	SSDZ1810006	Duck	Zhejiang	*S*.Typhimurium	2019.07.08
	SSDZ1810007	Duck	Zhejiang	*S*.Typhimurium	2019.07.08
	SSDZ1810008	Duck	Zhejiang	*S*.Typhimurium	2019.07.08
	SSDZ1810009	Duck	Zhejiang	*S*.Typhimurium	2019.07.08
	SSDZ1810010	Duck	Zhejiang	*S*.Typhimurium	2019.07.08
	SSDZ1810011	Duck	Zhejiang	*S*.Typhimurium	2019.07.08
	SSDZ1810012	Duck	Zhejiang	*S*.Typhimurium	2019.07.08
	SSDZ1810013	Duck	Zhejiang	*S*.Typhimurium	2019.07.08
	SSDZ1810014	Duck	Zhejiang	*S*.Typhimurium	2019.07.08
	SSDZ1810015	Duck	Zhejiang	*S*.Typhimurium	2019.07.08
	SSDZ1810016	Duck	Zhejiang	*S*.Typhimurium	2019.07.08
	SSDZ1810017	Duck	Zhejiang	*S*.Typhimurium	2019.07.08
	SSDZ1810018	Duck	Zhejiang	*S*.Typhimurium	2019.07.08
	SSDZ1810019	Duck	Zhejiang	*S*.Typhimurium	2019.07.08
	SSDZ1810020	Duck	Zhejiang	*S*.Typhimurium	2019.07.08
	SSDZ1810021	Duck	Zhejiang	*S*.Typhimurium	2019.07.08
	SSDZ1810022	Duck	Zhejiang	*S*.Typhimurium	2019.07.08
	SSDZ1810023	Duck	Zhejiang	*S*.Typhimurium	2019.07.08
	SSDZ1810024	Duck	Zhejiang	*S*.Typhimurium	2019.07.08
	SSDZ1810025	Duck	Zhejiang	*S*.Typhimurium	2019.07.08
	SSDZ1810026	Duck	Zhejiang	*S*.Typhimurium	2019.07.08
	SSDZ1810027	Duck	Zhejiang	*S*.Typhimurium	2019.07.08
	SSDZE1810002	Duck	Zhejiang	*S*.Typhimurium	2019.07.08
	SSDZE1810003	Duck	Zhejiang	*S*.Typhimurium	2019.07.08
	SSDZE1810004	Duck	Zhejiang	*S*.Typhimurium	2019.07.08
	SSDZE1810005	Duck	Zhejiang	*S*.Typhimurium	2019.07.08
	SSDZE1810006	Duck	Zhejiang	*S*.Typhimurium	2019.07.08
	SSDZE1810007	Duck	Zhejiang	*S*.Typhimurium	2019.07.08
	SSDZE1810008	Duck	Zhejiang	*S*.Typhimurium	2019.07.08
	SSPZ1804001	Pig	Zhejiang	*S*.Typhimurium	2019.07.08
	SSPZ1804002	Pig	Zhejiang	*S*.Typhimurium	2019.07.08
	SSPZ1804003	Pig	Zhejiang	*S*.Typhimurium	2019.07.08
	SSPZ1804004	Pig	Zhejiang	*S*.Typhimurium	2019.07.08
	SSPZ1804005	Pig	Zhejiang	*S*.Typhimurium	2019.07.08
	SSPZ1804006	Pig	Zhejiang	*S*.Typhimurium	2019.07.08
	SSPZ1804007	Pig	Zhejiang	*S*.Typhimurium	2019.07.08
	SSPZ1804008	Pig	Zhejiang	*S*.Typhimurium	2019.07.08
	SSPZ1804009	Pig	Zhejiang	*S*.Typhimurium	2019.07.08
	SSPZ18040010	Pig	Zhejiang	*S*.Typhimurium	2019.07.08

	SSPZ1804011	Pig	Zhejiang	*S.*Typhimurium	2019.07.08
	SSPZ1804012	Pig	Zhejiang	*S.*Typhimurium	2019.07.08
	SSPZ1804014	Pig	Zhejiang	*S.*Typhimurium	2019.07.08
	SSPZ1804015	Pig	Zhejiang	*S.*Typhimurium	2019.07.08
	SSPZ1804016	Pig	Zhejiang	*S.*Typhimurium	2019.07.08
	SSPZ1806001	Duck	Zhejiang	*S.*Typhimurium	2019.07.08
	ATCC700428	-	-	*S.*Typhimurium	-
14028	ATCC14028	-	-	*S.*Typhimurium	-
30	sm.20-30-MH	Chick	Shanxi	*S.* Enteritidis	2017.02.08
2C-4	SM2C-4.YS	Chick	Shandong	*S.* Enteritidis	2018.04.16
	SM.Y002-2G.H	Chick	Shandong	*S.* Enteritidis	2018.04.16
	SM.Y004G.H	Chick	Shandong	*S.* Enteritidis	2018.04.16
1-10	SM-J1-10-SX	Chick	Shanxi	*S.* Enteritidis	2018.05.21
4C.G	SM4C.G-MH	Chick	Shandong	*S.* Enteritidis	2017.09.01
21dh2	SM2C21dh2.MH	Chick	Shandong	*S.* Enteritidis	2018.04.11
21dh9	SM42C21dh9.MH	Chick	Shandong	*S.* Enteritidis	2018.04.11
23C04	SM23C04dh1.MH	Chick	Shandong	*S.* Enteritidis	2018.04.11
	SM30C21dh4.MH	Chick	Shandong	*S.* Enteritidis	2018.04.11
	SM30C21ds13.MH	Chick	Shandong	*S.* Enteritidis	2018.04.11
	SM39C21dh1.MH	Chick	Shandong	*S.* Enteritidis	2018.04.11
18090011	SSCZ18090011	Chick	Zhejiang	*S.* Enteritidis	2019.07.08
	SSCZ18090012	Chick	Zhejiang	*S.* Enteritidis	2019.07.08
	SSCZ18090013	Chick	Zhejiang	*S.* Enteritidis	2019.07.08
	SSCZ18090014	Chick	Zhejiang	*S.* Enteritidis	2019.07.08
18090015	SSCZ18090015	Chick	Zhejiang	*S.* Enteritidis	2019.07.08
	SSCZ18090016	Chick	Zhejiang	*S.* Enteritidis	2019.07.08
	SSCZ18090017	Chick	Zhejiang	*S.* Enteritidis	2019.07.08
	SSCZ18090018	Chick	Zhejiang	*S.* Enteritidis	2019.07.08
	SSCZ18090019	Chick	Zhejiang	*S.* Enteritidis	2019.07.08
18090020	SSCZ18090020	Chick	Zhejiang	*S.* Enteritidis	2019.07.08
	SSDZE1810001	Duck	Zhejiang	*S.* Enteritidis	2019.07.08
004X.H	SM.Y004X.H	duck	Shandong	*S.* Enteritidis	2018.04.19
10#-DL	SM-10#-DL	Chick	Shandong	*S.* Enteritidis	2021.09.12
13076	ATCC13076	-	-	*S.* Enteritidis	-
	SM-11-1	Chick	Shandong	*S.* Enteritidis	2021.09.12
	SM-16-1	Chick	Shandong	*S.* Enteritidis	2021.09.12
	SM.C11.TA	Chick	Shandong	*S.* Enteritidis	2018.5.10
	SM.D96.TA	Duck	Shandong	*S.* Enteritidis	2018.5.10
	SM-3-10-SX	Chick	Shanxi	*S.* Enteritidis	2018.05.21
	SM-JP1-66-SX	Chick	Shanxi	*S.* Enteritidis	2018.05.21
	SM-JP1-75-SX	Chick	Shanxi	*S.* Enteritidis	2018.05.21
	SM-SP1	Chick	Shandong	*S.* Pullorum	2019.5.13
	SM-SP2	Chick	Shandong	*S.* Pullorum	2019.5.13

	SM-SP3	Chick	Shandong	*S.* Pullorum	2019.5.13
	E. coli-1	Chick	Shandong	*E. coli*	2013.01.04
	E. coli-2	Chick	Shandong	*E. coli*	2013.01.04
	E. coli-3	Chick	Shandong	*E. coli*	2013.01.04
	E. coli-4	Chick	Shandong	*E. coli*	2013.01.04
	E. coli-5	Chick	Shandong	*E. coli*	2013.01.04
	E. coli-6	Chick	Shandong	*E. coli*	2013.01.04
	E. coli-7	Chick	Shandong	*E. coli*	2013.01.04
	E. coli-8	Chick	Shandong	*E. coli*	2013.01.04
	E. coli-9	Chick	Shandong	*E. coli*	2013.01.04
	E. coli-10	Chick	Shandong	*E. coli*	2013.01.04
	KP-1	Chick	Shandong	*K. pneumoniae*	2018.09.01
	KP-2	Chick	Shandong	*K. pneumoniae*	2018.09.01
	KP-3	Chick	Shandong	*K. pneumoniae*	2018.09.01
	KP-4	Chick	Shandong	*K. pneumoniae*	2018.09.01
	KP-5	Chick	Shandong	*K. pneumoniae*	2018.09.01
	KP-6	Chick	Shandong	*K. pneumoniae*	2018.09.01
	KP-7	Chick	Shandong	*K. pneumoniae*	2018.09.01
	KP-8	Chick	Shandong	*K. pneumoniae*	2018.09.01
	KP-9	Chick	Shandong	*K. pneumoniae*	2018.09.01
	KP-10	Chick	Shandong	*K. pneumoniae*	2018.09.01
	E-1	Chick	Shandong	Enterococcus	2018.09.01
	E-2	Chick	Shandong	Enterococcus	2018.09.01
	E-3	Chick	Shandong	Enterococcus	2018.09.01
	E-4	Chick	Shandong	Enterococcus	2018.09.01
	E-5	Chick	Shandong	Enterococcus	2018.09.01
	E-6	Chick	Shandong	Enterococcus	2018.09.01
	E-7	Chick	Shandong	Enterococcus	2018.09.01
	E-8	Chick	Shandong	Enterococcus	2018.09.01
	E-9	Chick	Shandong	Enterococcus	2018.09.01
	E-10	Chick	Shandong	Enterococcus	2018.09.01
	PA-1	Chick	Shandong	*P.aeruginosa*	2018.09.01
	PA-2	Chick	Shandong	*P.aeruginosa*	2018.09.01
	PA-3	Chick	Shandong	*P.aeruginosa*	2018.09.01
	PA-4	Chick	Shandong	*P.aeruginosa*	2018.09.01
	PA-5	Chick	Shandong	*P.aeruginosa*	2018.09.01
	PA-6	Chick	Shandong	*P.aeruginosa*	2018.09.01
	PA-7	Chick	Shandong	*P.aeruginosa*	2018.09.01
	PA-8	Chick	Shandong	*P.aeruginosa*	2018.09.01
	PA-9	Chick	Shandong	*P.aeruginosa*	2018.09.01
	PA-10	Chick	Shandong	*P.aeruginosa*	2018.09.01

Table S2. Predicted ORFs in the genome of phage PJN012

Qeury name	Qeury length	start	stop	strand	Predictive function [Closest hit]	Query Cover	E-values	Identity %	Accession number
ORF1	105	2	106	+	hypothetical protein [Salmonella phage Shelanagig]	97%	5e-16	100	YP_010747286.1
ORF2	390	151	540	+	hypothetical protein QA057_gp46 [Salmonella phage SLMP1]	99%	5e-89	100	YP_010748145.1
ORF3	702	731	1432	+	hypothetical protein K9_008 [Salmonella phage Kenya-K9]	99%	3e-128	99.57	WCZ56575.1
ORF4	1050	1436	2485	+	putative coat protein [Salmonella phage vB_SenS_S532]	95%	0.0	100	YP_010746857.1
ORF5	288	2546	2833	+	putative head protein [Salmonella phage vB-SeS-01]	98%	1e-41	98.95	UXQ84698.1
ORF6	351	2845	3195	+	Hoc-like head decoration [Salmonella phage vB_SenS-Ent1]	99%	3e-76	100	YP_007010465.1
ORF7	189	3232	3420	+	head-tail joining protein [Salmonella phage vB_SenS-EnJE1]	98%	3e-35	100	YP_010746810
ORF8	510	3424	3933	+	head-tail joining protein [Salmonella phage vB_SenS-EnJE1]	99%	8e-119	100	YP_010746809.1
ORF9	606	3936	4541	+	neck protein [Salmonella phage BPS11Q3]	99%	9e-140	100	YP_009322868.1
ORF10	360	4541	4900	+	tail protein [Salmonella phage vB_SenS_S532]	99%	2e-79	100	YP_010746863.1

ORF11	396	4897	5292	+	hypothetical protein QA019_gp49 [Salmonella phage SE-W109]	99%	3e-73	98.47	YP_010745813.1
ORF12	420	5292	5711	+	hypothetical protein [Salmonella phage vB-SeS-01]	99%	2e-96	100	UXQ84691.1
ORF13	1170	5711	6880	+	putative tail protein [Salmonella phage vB_SenS_S532]	99%	0.0	100	YP_010746866.1
ORF14	672	7583	6912	-	putative DNA-binding protein [Salmonella phage vB_SenS_S532]	99%	4e-163	99.55	YP_010746867.1
ORF15	231	7927	7697	-	hypothetical protein [Salmonella phage GSP003]	98%	7e-48	100	UXE05702.1
ORF16	1131	9054	7924	-	oxidoreductase [Salmonella phage vB_SenS-EnJE1]	99%	0.0	100	YP_010746801.1
ORF17	180	9296	9117	-	superinfection immunity protein [Pseudomonadota]	98%	8e-32	100	WP_015984947.1
ORF18	417	9466	9882	+	phage tail assembly chaperone [Bacteria]	99%	9e-97	100	WP_064624761.1
ORF19	360	9888	10247	+	hypothetical protein [Pseudomonadota]	99%	2e-82	99.16	WP_016062378.1
ORF20	2334	10240	12573	+	tape measure protein [Salmonella phage vB_SenS_S532]	99%	0.0	99.87	YP_010746873.1
ORF21	444	12632	13075	+	hypothetical protein QA037_gp48 [Salmonella phage vB_SenS_S532]	99%	8e-105	99.32	YP_010746874.1
ORF22	516	13072	13587	+	minor tail protein [Salmonella phage wksl3]	99%	3e-122	100	YP_009608662.1

ORF23	366	135<br>84	1394<br>9	+	hypothetical protein QA018_gp16 [Salmonella phage SS5]	99%	1e-84	99.17	YP_0107457<br>13.1
ORF24	2559	139<br>40	1649<br>8	+	hypothetical protein [Salmonellam phagevB_SenS_ER 13]	99%	0.0	99.06	QQO87402.<br>1
ORF25	2031	165<br>11	1854<br>1	+	tailspike protein [Salmonella phage vB_SalS_ABTNLsp 11241]	99%	0.0	98.96	QXH32880.<br>1
ORF26	162	187<br>87	1862<br>6	-	hypothetical protein [Salmonella enterica]	98%	2e-28	100	WP_170869<br>161.1
ORF27	2469	212<br>52	1878<br>4	-	DNA helicase [Salmonella phage vB_StyS-sam]	99%	0.0	98.3	YP_0107461<br>96.1
ORF28	498	217<br>46	2124<br>9	-	hypothetical protein QA036_gp19 [Salmonella phage vB_SenS-EnJE1]	99%	2e-106	100	YP_0107467<br>91.1
ORF29	192	219<br>39	2174<br>8	-	hypothetical protein QA037_gp55 [Salmonella phage vB_SenS_S532]	98%	3e-36	100	YP_0107468<br>81.1
ORF30	288	222<br>58	2197<br>1	-	putative restriction endonuclease [Salmonella phage vB_SenS-EnJE1]	98%	5e-62	100	YP_0107467<br>89.1
ORF31	3099	254<br>43	2234<br>5	-	intein-containing DNA polymerase precursor [Salmonella phage vB_SenS_S528]	99%	0.0	99.61	YP_0107470<br>13.1
ORF32	627	261<br>27	2550<br>1	-	DUF2815 family protein [Salmonella phage SETP3]	99%	0.0	100	YP_0011108<br>10.1
ORF33	1275	274<br>83	2620<br>9	-	DUF2800 domain-containing protein	99%	0.0	99.76	UXQ84737.<br>1

					[Salmonella phage vB-SeS-01]				
ORF34	513	28037	27525	-	hypothetical protein QA036_gp13 [Salmonella phage vB_SenS-EnJE1]	99%	2e-76	99.41	YP_010746785.1
ORF35	219	28171	28389	+	helix-turn-helix transcriptional regulator [Salmonella enterica]	98%	6e-45	100	WP_015984911.1
ORF36	2187	30589	28403	-	primase/helicase [Salmonella phage vB_SenS_phi135]	99%	0.0	99.04	AYP69663.1
ORF37	234	30879	30646	-	putative UvsX-like protein [Salmonella phage vB_SenS_S532]	98%	9e-47	98.7	YP_010746891.1
ORF38	171	31046	30876	-	DNA-binding protein [Salmonella phage vB_SenS-EnJE1]	98%	4e-33	100	YP_010746782.1
ORF39	180	31749	31928	+	hypothetical protein QA023_gp01 [Salmonella phage wast]	76%	3e-23	100	YP_010746013.1
ORF40	165	32049	32213	+	hypothetical protein W71701E2_4 [Salmonella phage PIZ SAE-01E2]	98%	3e-31	98.15	QGZ13164.1
ORF41	114	32277	32390	+	hypothetical protein BOX12_gp27 [Salmonella phage BPS11Q3]	97%	1e-17	97.3	YP_009322834.1
ORF42	498	32383	32880	+	hypothetical protein [Salmonella phage vB-SeS-01]	99%	1e-118	98.79	UXQ84727.1
ORF43	204	32877	33080	+	hypothetical protein SaPhSE2_gp18	98%	4e-42	100	YP_005098114.1

					[Salmonella phage SE2]				
ORF44	372	33083	33454	+	hypothetical protein DTE88_23115 [Salmonella enterica subsp. enterica serovar Enteritidis]	99%	8e-68	98.37	EBX7899592.1
ORF45	435	33460	33894	+	hypothetical protein [Salmonella phage vB_SpuP_Spp11]	99%	2e-88	98.61	QIG57315.1
ORF46	282	33973	34254	+	holin [Salmonella phage f18SE]	98%	5e-60	100	YP_009191600.1
ORF47	291	34256	34546	+	putative class II holin [Salmonella phage vB_SenS_S532]	98%	1e-62	100	YP_010746836.1
ORF48	489	34524	35012	+	lysin [Salmonella phage vB_SenS-EnJE6]	99%	1e-87	98.15	YP_010747086.1
ORF49	186	35199	35384	+	hypothetical protein QA060_gp07 [Salmonella phage T102]	98%	2e-36	96.72	YP_010748268.1
ORF50	156	35381	35536	+	DUF2737 family protein [Salmonella phage SE2]	98%	1e-28	100	YP_005098120.1
ORF51	183	35533	35715	+	putative NinZ-like protein [Salmonella phage SS5]	96%	4e-33	98.31	YP_010745747.1
ORF52	150	35712	35861	+	hypothetical protein W71701E2_16 [Salmonella phage PIZ SAE-01E2]	98%	7e-24	97.96	QGZ13176.1
ORF53	225	35858	36082	+	NinH family protein [Salmonella phage SETP3]	98%	1e-46	100	YP_008859658.1
ORF54	120	36227	36346	+	hypothetical protein QA037_gp19	97%	2e-17	97.44	YP_010746845.1

					[Salmonella phage vB_SenS_S532]				
ORF55	183	36381	36563	+	hypothetical protein BI170_gp08 [Salmonella phage MA12]	98%	2e-33	98.33	YP_009280136.1
ORF56	132	36624	36755	+	hypothetical protein SaPhSE2_gp29 [Salmonella phage SE2]	97%	2e-22	100	YP_005098125.1
ORF57	507	36784	37290	+	putative terminase small subunit [Salmonella phage GSP162]	99%	1e-91	99.4	UUT41002.1
ORF58	1272	37280	38551	+	terminase large subunit [Salmonella phage vB_SenS_S528]	99%	0.0	99.76	YP_010746977.1
ORF59	1476	38564	40039	+	62kDa structural protein [Salmonella phage vB_SenS_S528]	98%	0.0	100	YP_010746978.1
ORF60	651	40720	40070	-	hypothetical protein QA042_gp37 [Salmonella phage vB_SalS-S10]	99%	7E-160	100	YP_010747194.1
ORF61	1044	40886	41929	+	head morphogenesis protein [Salmonella phage vB_SenS-EnJE1]	99%	0.0	99.42	YP_010746817.1
ORF62	459	41932	42390	+	neck whiskers protein [Salmonella phage vB_SenS-EnJE1]	99%	6E-86	99.34	YP_010746816.1

Table S3. Predicted ORFs in the genome of phage PJN042.

Qeury name	Qeury length	start	stop	strand	Predictive function [Closest hit]	Query Cover	E-values	Identity %	Accession number
ORF1	417	454	38	-	DNA binding protein [Salmonella phage MET_P1_137_112]	99%	4e-80	99.28	WFG41354.1
ORF2	252	992	741	-	hypothetical protein [Salmonella phage Stitch]	98%	5e-52	98.80	YP_009145948.1
ORF3	210	1368	1159	-	hypothetical protein [Salmonella phage Seafire]	97%	4e-40	98.53	YP_009816657.1
ORF4	159	1523	1365	-	hypothetical protein [Salmonella phage Seafire]	98%	4e-30	100.00	YP_009816658.1
ORF5	1002	2601	3602	+	hypothetical protein [Salmonella phage JNwz02]	99%	0.0	100.00	QYC50602.1
ORF6	495	3667	4161	+	hypothetical protein [Salmonella phage JNwz02]	99%	7e-102	100.00	QYC50603.1
ORF7	228	4269	4496	+	hypothetical protein [Salmonella phage LVR16A]	98%	1e-47	100.00	YP_009804297.1
ORF8	213	4550	4762	+	hypothetical protein [Salmonella phage JNwz02]	98%	2e-42	100.00	QYC50605.1
ORF9	345	4872	5216	+	hypothetical protein [Salmonella phage GSP001]	99%	6e-77	98.25	UUT40839.1
ORF10	201	5213	5413	+	hypothetical protein [Escherichia phage Eps7]	98%	1e-39	100.00	YP_001836937.1
ORF11	96	5540	5635	+	hypothetical protein [Salmonella phage STG2]	96%	7e-13	100.00	YP_009815113.1
ORF12	195	5651	5845	+	hypothetical protein HOV06_gp013 [Salmonella phage 1-23]	98%	5e-38	100.00	YP_009819290.1

ORF13	177	6806	6630	-	hypothetical protein HWC67_gp013 [Salmonella phage 1-29]	83%	6e-15	77.55	YP_009852207.1
ORF14	711	8101	7391	-	hypothetical protein PJM38_0017 [Salmonella phage vB_SenS_UTK0010]	99%	3e-170	99.58	WDR22263.1
ORF15	390	8493	8104	-	hypothetical protein BSP22A_0022 [Salmonella phage BSP22A]	99%	3e-86	99.22	ARM69685.1
ORF16	663	9218	8556	-	hypothetical protein PJM38_0019 [Salmonella phage vB_SenS_UTK0010]	99%	2e-161	98.64	WDR22265.1
ORF17	186	9403	9218	-	hypothetical protein SPThor_129 [Salmonella phage SP_Thor]	64%	7e-05	100.00	QQV93652.1
ORF18	234	9636	9403	-	hypothetical protein CPT_Stitch19 [Salmonella phage Stitch]	98%	5e-31	100.00	YP_009145960.1
ORF19	543	10389	9847	-	hypothetical protein CPT_Stitch20 [Salmonella phage Stitch]	99%	7e-121	98.89	YP_009145961.1
ORF20	252	10661	10410	-	capsid and scaffold protein [Salmonella phage OSY-STA]	98%	1e-49	98.80	YP_009851855.1
ORF21	468	11202	10735	-	hypothetical protein CPT_Stitch22 [Salmonella phage Stitch]	99%	7e-112	100.00	YP_009145963.1
ORF22	201	11399	11199	-	hypothetical protein CPT_Stitch23 [Salmonella phage Stitch]	98%	5e-41	100.00	YP_009145964.1
ORF23	333	11832	11500	-	hypothetical protein BOW73_gp028 [Salmonella phage 100268_sal2]	99%	2e-73	99.09	YP_009320752.1
ORF24	246	120	1182	-	hypothetical protein	98%	1e-48	100.00	YP_00981216

		67	2		HOU17_gp029 [Salmonella phage Sw2]				5.1
ORF25	282	12345	12064	-	hypothetical protein BD13_174 [Salmonella phage BD13]	98%	4e-41	98.92	UQT65314.1
ORF26	408	12749	12342	-	hypothetical protein HOU17_gp031 [Salmonella phage Sw2]	99%	6e-75	100.00	YP_009812167.1
ORF27	252	13000	12749	-	hypothetical protein CPT_Stitch28 [Salmonella phage Stitch]	98%	1e-51	100.00	YP_009145969.1
ORF28	432	13510	13079	-	hypothetical protein BD13_171 [Salmonella phage BD13]	99%	2e-100	100.00	UQT65311.1
ORF29	102	13679	13578	-	hypothetical protein	-			-
ORF30	585	14256	13672	-	putative serine/threonine protein phosphatase [Salmonella phage JNwz02]	99%	2e-137	98.45	QYC50625.1
ORF31	207	14462	14256	-	Rz-like spanin [Salmonella phage SH9]	98%	4e-41	100.00	YP_009804249.1
ORF32	369	14830	14462	-	hypothetical protein HOT60_gp113 [Salmonella phage S114]	99%	1e-82	99.18	YP_009805081.1
ORF33	864	15693	14830	-	serine/threonine protein phosphatase [Salmonella enterica subsp. enterica]	99%	0.0	99.30	EDY0344169.1
ORF34	381	16073	15693	-	hypothetical protein BOW73_gp039 [Salmonella phage 100268_sal2]	99%	2e-49	98.41	YP_009320763.1
ORF35	291	16465	16175	-	thioredoxin [Salmonella phage Stitch]	98%	2e-64	100.00	YP_009145977.1

ORF36	411	16868	16458	-	hypothetical protein BOW73_gp041 [Salmonella phage 100268_sal2]	99%	8e-83	100.00	YP_009320765.1
ORF37	417	17360	16944	-	hypothetical protein BOW73_gp042 [Salmonella phage 100268_sal2]	99%	9e-95	100.00	YP_009320766.1
ORF38	414	17849	17436	-	endolysin [Salmonella phage Stitch]	99%	1e-95	99.27	YP_009145980.1
ORF39	657	18502	17846	-	holin [Salmonella phage 100268_sal2]	99%	5e-158	99.54	YP_009320768.1
ORF40	600	19258	18659	-	ATP-dependent protease [Salmonella phage rokbiter]	99%	2e-147	98.99	YP_009858352.1
ORF41	753	20023	19271	-	dNMP kinase [Salmonella phage vB_SenS_SB9]	99%	0.0	99.60	QDH47346.1
ORF42	450	20756	20307	-	hypothetical protein BFINDDAI_00041 [Salmonella phage EH2]	99%	3e-90	98.66	WMT11141.1
ORF43	699	21411	20713	-	hypothetical protein HWD25_gp128 [Salmonella phage fuchur]	99%	2e-171	99.57	YP_009858513.1
ORF44	348	21904	21557	-	hypothetical protein HOU17_gp049 [Salmonella phage Sw2]	99%	8e-78	100.00	YP_009812185.1
ORF45	285	22305	22021	-	hypothetical protein HOT62_gp122 [Salmonella phage S126]	98%	2e-48	98.94	YP_009805387.1
ORF46	420	22968	22549	-	hypothetical protein vBSenS3_61 [Salmonella phage vB_SenS-3]	99%	2e-88	99.28	QIN93389.1
ORF47	300	23260	22961	-	hypothetical protein CPT_Stitch50 [Salmonella phage Stitch]	99%	8e-65	100.00	YP_009145991.1

ORF48	282	23534	23253	-	hypothetical protein HOS37_gp044 [Escherichia phage saus132]	92%	1e-55	100.00	YP_009794879.1
ORF49	396	24006	23611	-	hypothetical protein CPT_Stitch52 [Salmonella phage Stitch]	99%	3e-90	100.00	YP_009145993.1
ORF50	186	24250	24065	-	hypothetical protein HOT53_gp135 [Salmonella phage SH9]	98%	1e-24	100.00	YP_009804230.1
ORF51	300	24614	24315	-	hypothetical protein [Citrobacter portucalensis]	99%	1e-67	98.99	WP_319846364.1
ORF52	369	25064	24696	-	pyruvate formate-lyase [Salmonella phage S113]	99%	4e-83	100.00	YP_009804895.1
ORF53	207	25890	25684	-	hypothetical protein [Salmonella phage SP76]	98%	1e-41	100.00	QPI16039.1
ORF54	294	26183	25890	-	hypothetical protein [Salmonella phage SP76]	98%	5e-64	100.00	QPI16038.1
ORF55	165	26507	26343	-	hypothetical protein [Salmonella phage SP76]	98%	1e-28	100.00	QPI16037.1
ORF56	222	26721	26500	-	hypothetical protein BD13_140 [Salmonella phage BD13]	98%	2e-46	100.00	UQT65283.1
ORF57	201	27184	26984	-	hypothetical protein [Salmonella phage SE3]	98%	5e-39	96.97	QEG07565.1
ORF58	204	27940	27737	-	hypothetical protein GECvBN7_gp080c [Salmonella phage GEC_vB_N7]	98%	1e-41	100.00	QPI15523.1
ORF59	180	28305	28126	-	hypothetical protein CPT_Stitch62 [Salmonella phage Stitch]	98%	1e-34	100.00	YP_009146003.1
ORF60	219	28628	28410	-	hypothetical protein HWC65_gp159	98%	2e-32	100.00	YP_009851971.1

					[Salmonella phage 2-3]				
ORF61		29422	29069	-	hypothetical protein HOT53_gp144 [Salmonella phage SH9]	99%	3e-79	100	YP_009804221.1
ORF62	318	29972	29655	-	hypothetical protein BD13_127 [Salmonella phage BD13]	99%	1e-72	100.00	UQT65277.1
ORF63	165	30235	30071	-	hypothetical protein BOW73_gp177 [Salmonella phage 100268_sal2]	98%	2e-30	100.00	YP_009320796.1
ORF64	186	31356	31171	-	hypothetical protein CPT_Stitch68 [Salmonella phage Stitch]	98%	9e-33	98	YP_009146009.1
ORF65	519	32032	31514	-	hypothetical protein HWD22_gp147 [Salmonella phage bombadil]	99%	2e-111	99	YP_009857996.1
ORF66	273	32404	32132	-	hypothetical protein HOS37_gp151 [Escherichia phage saus132]	98%	7e-59	98	YP_009794897.1
ORF67	273	32726	32454	-	hypothetical protein BOW73_gp171 [Salmonella phage 100268_sal2]	98%	3e-60	98	YP_009320802.1
ORF68	276	33550	33275	-	hypothetical protein HOS37_gp149 [Escherichia phage saus132]	98%	4e-59	98	YP_009794899.1
ORF69	207	33846	33640	-	hypothetical protein [Salmonella phage JNwz02]	98%	9e-40	98	QYC50503.1
ORF70	123	33982	33860	-	hypothetical protein CPT_Stitch74 [Salmonella phage Stitch]	97%	1e-20	97	YP_009146015.1
ORF71	339	34421	34083	-	hypothetical protein BD13_109 [Salmonella phage	99%	1e-59	99	UQT65268.1

					BD13]				
ORF72	186	34608	34423	-	hypothetical protein BD13_108 [Salmonella phage BD13]	98%	6e-37	98	UQT65267.1
ORF73	189	34805	34617	-	hypothetical protein HOS37_gp144 [Escherichia phage saus132]	98%	6e-36	98	YP_009794904.1
ORF74	510	35506	34997	-	putative HNH homing endonuclease [Salmonella phage Seafire]	99%	3e-123	99	YP_009816733.1
ORF75	297	35873	35577	-	hypothetical protein BD13_103 [Salmonella phage BD13]	98%	2e-65	98	UQT65264.1
ORF76	678	36657	35980	-	PnuC-like nicotinamide mononucleotide transport [Escherichia phage saus132]	99%	3e-142	100.00	YP_009794907.1
ORF77	1056	37714	36659	-	transcriptional regulator [Salmonella phage BD13]	99%	0.0	100.00	UQT65262.1
ORF78	945	38930	37986	-	hypothetical protein GRN08_0870 [Salmonella phage GRNsp8]	99%	0.0	99.68	URG17751.1
ORF79	510	39556	39047	-	hypothetical protein [Salmonella phage STWB21]	99%	1e-105	99.41	QTJ63331.1
ORF80	171	41336	41166	-	hypothetical protein AGC_0090 [Escherichia phage Eps7]	98%	5e-30	100.00	YP_001837013.1
ORF81	450	41854	41405	-	endolysin [Salmonella phage vB_SenS_SB13]	99%	2e-106	100.00	YP_009848091.1
ORF82	318	42177	41860	-	hypothetical protein BOW73_gp155	99%	8e-71	100.00	YP_009320818.1

					[Salmonella phage 100268_sal2]				
ORF83	639	43255	42617	-	tail fiber protein [Salmonella phage bux]	99%	1e-148	99	QIQ61610.1
ORF84	183	43491	43309	-	hypothetical protein AGC_0095 [Escherichia phage Eps7]	98%	2e-33	100.00	YP_001837018.1
ORF85	702	44263	43562	-	exonuclease [Salmonella phage Sw2]	99%	3e-175	100.00	YP_009812233.1
ORF86	213	44506	44294	-	hypothetical protein CPT_Stitch91 [Salmonella phage Stitch]	98%	7e-43	100.00	YP_009146032.1
ORF87	216	44763	44548	-	tail length tape-measure protein [Salmonella phage atrejo]	98%	5e-40	98.59	YP_009858972.1
ORF88	516	45340	44825	-	hypothetical protein HOS37_gp128 [Escherichia phage saus132]	91%	7e-112	100.00	YP_009794920.1
ORF89	279	45702	45424	-	hypothetical protein [Citrobacter portucalensis]	99%	8e-79	99.15	WP_319846371.1
ORF90	477	46255	45779	-	Rnase H [Escherichia phage Eps7]	99%	2e-113	99.37	YP_001837024.1
ORF91	270	46524	46255	-	hypothetical protein [Citrobacter portucalensis]	98%	3e-56	100.00	WP_015989765.1
ORF92	255	46778	46524	-	hypothetical protein CPT_Stitch98 [Salmonella phage Stitch]	98%	1e-42	98.81	YP_009146039.1
ORF93	855	47725	46871	-	thymidylate synthase [Salmonella phage S113]	99%	0.0	99.30	YP_009804850.1
ORF94	531	48252	47722	-	dihydrofolate reductase [Salmonella phage	99%	2e-115	100.00	YP_009848076.1

					vB_SenS_SB13]				
ORF95	1146	49397	48252	-	aerobic ribonucleoside diphosphate reductase, beta subunit [Salmonella phage Sepoy]	99%	0.0	100.00	YP_009845314.1
ORF96	2439	51942	49504	-	aerobic ribonucleoside diphosphate reductase, large subunit [Salmonella phage Sw2]	96%	0.0	99.87	YP_009812246.1
ORF97	243	52201	51959	-	tail length tape measure protein [Salmonella phage Sw2]	98%	2e-50	100.00	YP_009812247.1
ORF98	753	52955	52203	-	PhoH-like phosphate starvation-inducible protein [Salmonella phage Stitch]	99%	0.0	100.00	YP_009146045.1
ORF99	1875	53308	55182	+	ribonucleotide reductase of class III (anaerobic), large subunit [Salmonella phage SP76]	98%	7e-62	100.00	QPI15992.1
ORF100	282	55281	55562	+	hypothetical protein bas26_0055 [Escherichia phage GreteKellenberger]	98%	7e-62	100.00	QXV79662.1
ORF101	204	55572	55775	+	hypothetical protein HOT62_gp055 [Salmonella phage S126]	98%	6e-38	98.51	YP_009805333.1
ORF102	843	55938	56780	+	Sir2 (NAD-dependent deacetylase) [Salmonella phage Sepoy]	99%	0.0	99.64	YP_009845323.1
ORF103	216	56767	56982	+	hypothetical protein HOU17_gp119 [Salmonella phage Sw2]	98%	5e-44	97.18	YP_009812254.1

ORF104	186	56979	57164	+	hypothetical protein HOR05_gp030 [Escherichia phage phiAPCEc03]	98%	9e-33	98.36	YP_009785314.1
ORF105	507	57151	57657	+	Sir2 (NAD-dependent deacetylase) [Salmonella phage bombadil]	99%	2e-108	98.21	YP_009857953.1
ORF106	429	57660	58088	+	hypothetical protein HOT67_gp049 [Salmonella phage S124]	99%	2e-99	100.00	YP_009806101.1
ORF107	396	58098	58493	+	hypothetical protein BOW73_gp128 [Salmonella phage 100268_sal2]	99%	4e-75	99.24	YP_009320845.1
ORF108	2790	59110	61899	+	replication origin binding protein [Salmonella phage BD13]	99%	0.0	100.00	UQT65231.1
ORF109	234	61883	62116	+	hypothetical protein AGC_0124 [Escherichia phage Eps7]	98%	3e-47	100.00	YP_001837047.1
ORF110	705	62188	62892	+	hypothetical protein NU751_004521, partial [Salmonella enterica]	99%	2e-170	100.00	EJO9850529.1
ORF111	234	62885	63118	+	transcriptional regulator [Salmonella phage 1-29]	98%	7e-47	98.70	YP_009852303.1
ORF112	411	63226	63636	+	DNA binding protein [Salmonella phage 100268_sal2]	99%	2e-66	99.26	YP_009320850.1
ORF113	297	63673	63969	+	hypothetical protein AGC_0128 [Escherichia phage Eps7]	98%	5e-51	100.00	YP_001837051.1
ORF114	309	64020	64328	+	transcriptional regulator protein [Salmonella phage vaffelhjerte]	99%	2e-69	100.00	QIN99677.1

ORF115	201	64414	64614	+	hypothetical protein [Salmonella phage JNwz02]	98%	9e-39	100.00	QYC50550.1
ORF116	972	64607	65578	+	NAD-dependent DNA ligase subunit A [Salmonella phage polluks]	99%	0.0	99.69	QIO00329.1
ORF117	780	65781	66560	+	NAD-dependent DNA ligase [Salmonella phage S113]	99%	0.0	100.00	YP_009804827.1
ORF118	768	66553	67320	+	D5 protein [Salmonella phage faergetype]	93%	4e-171	100.00	YP_009858105.1
ORF119	1524	67352	68875	+	hypothetical protein HOT59_gp037 [Salmonella phage S113]	99%	0.0	100.00	YP_009804825.1
ORF120	891	68872	69762	+	DNA primase [Salmonella phage Seafire]	99%	0.0	100.00	YP_009816784.1
ORF121	2568	69825	72392	+	DNA polymerase [Salmonella phage BD13]	99%	0.0	99.88	UQT65218.1
ORF122	498	72385	72882	+	hypothetical protein BD13_55 [Salmonella phage BD13]	99%	2e-115	99.39	UQT65217.1
ORF123	1353	72879	74231	+	helicase [Salmonella phage Stitch]	99%	0.0	99.78	YP_009146071.1
ORF124	363	74372	74734	+	hypothetical protein BD13_53 [Salmonella phage BD13]	99%	2e-80	100.00	UQT65215.1
ORF125	774	74727	75500	+	hypothetical protein BD13_52 [Salmonella phage BD13]	99%	2e-177	100.00	UQT65214.1
ORF126	978	75540	76517	+	recombinase [Salmonella phage BD13]	99%	0.0	100.00	UQT65213.1
ORF127	1797	76540	78336	+	recombination related exonuclease [Salmonella phage	99%	0.0	100.00	YP_009848035.1

					vB_SenS_SB13]				
ORF128	483	78340	78822	+	hypothetical protein AGP59_23490 [Salmonella enterica subsp. enterica serovar Derby]	99%	3e-115	100.00	EDA1231026.1
ORF129	876	78822	79697	+	flap endonuclease [Escherichia phage Eps7]	99%	0.0	100.00	YP_001837066.1
ORF130	447	79694	80140	+	dUTPase [Salmonella phage S114]	91%	4e-97	100.00	YP_009804978.1
ORF131	2952	83610	80659	-	putative tail protein [Salmonella phage BD13]	99%	0.0	100.00	UQT65207.1
ORF132	423	84032	83610	-	collar tail protein for L-shaped tail fibre attachment [Escherichia phage Eps7]	99%	2e-97	100.00	YP_001837070.1
ORF133	2058	86095	84038	-	straight fibre tail protein [Salmonella phage 1-19]	99%	0.0	99.71	YP_009853090.1
ORF134	2850	88945	86096	-	tail protein [Salmonella phage BD13]	99%	0.0	100.00	UQT65204.1
ORF135	615	89556	88942	-	distal tail protein [Salmonella phage S132]	99%	1e-144	99.51	YP_009805597.1
ORF136	3711	93375	89665	-	tail length tape measure protein [Salmonella phage 3-29]	99%	0.0	99.92	YP_009819007.1
ORF137	369	93824	93456	-	tail assembly chaperone [Salmonella phage Sw2]	99%	8e-84	99.18	YP_009812293.1
ORF138	405	94290	93886	-	Tail assembly chaperone [Escherichia phage Eps7]	99%	1e-93	100.00	YP_001837077.1
ORF139	900	95186	94287	-	minor tail protein [Salmonella phage BSP22A]	99%	0.0	99.67	ARM69820.1

ORF140	1410	96600	95191	-	major tail protein [Salmonella phage Stitch]	99%	0.0	100.00	YP_009146090.1
ORF141	486	97112	96627	-	tail terminator protein [Salmonella phage Stitch]	99%	4e-116	100.00	YP_009146091.1
ORF142	768	97883	97116	-	tail completion or Neck1 protein [Salmonella phage Stitch]	99%	0.0	99.61	YP_009146092.1
ORF143	513	98395	97883	-	head-tail adaptor [Escherichia phage saus132]	99%	1e-123	100.00	YP_009794978.1
ORF144	1377	99831	98455	-	major capsid protein [Salmonella phage EH3]	99%	0.0	99.78	WMM35102.1
ORF145	633	100481	99849	-	head maturation protease [Escherichia phage Eps7]	99%	8e-154	100.00	YP_001837084.1
ORF146	483	100967	100485	-	Hoc-like head decoration [Salmonella phage S114]	99%	2e-111	100.00	YP_009804961.1
ORF147	1218	102181	100964	-	portal protein [Salmonella phage S113]	99%	0.0	100.00	YP_009804793.1
ORF148	438	102618	102181	-	hypothetical protein HOT60_gp003 [Salmonella phage S114]	99%	6e-90	99.31	YP_009804959.1
ORF149	1317	104049	102733	-	terminase large subunit [Salmonella phage Stitch]	99%	0.0	100.00	YP_009146099.1
ORF150	483	104531	104049	-	terminase small subunit [Escherichia phage Eps7]	99%	1e-86	99.38	YP_001837089.1
ORF151	1782	106323	104542	-	receptor-binding tail tip protein [Salmonella phage GSP001]	99%	0.0	99.66	UUT40857.1
ORF152	267	106407	106673	+	Cor superinfection exclusion protein [Salmonella phage	98%	5e-50	98.86	YP_009848213.1

					vB_SenS_SB13]				
ORF153	315	106748	107062	+	hypothetical protein bas26_0207 [Escherichia phage GreteKellenberger]	99%	4e-67	100.00	QXV79784.1
ORF154	105	107135	107239	+	hypothetical protein AGC_0170 [Escherichia phage Eps7]	57%	2e-04	100.00	YP_001837092.1
ORF155	195	107239	107433	+	hypothetical protein CPT_Stitch163 [Salmonella phage Stitch]	70%	5e-23	100.00	YP_009146104.1
ORF156	228	107426	107653	+	putative membrane protein [Escherichia phage PSa2]	76%	1e-17	100.00	UIR90742.1
ORF157	735	108546	107812	-	deoxynucleoside-5'-monophosphatase [Salmonella phage BD13]	99%	0.0	100.00	UQT65181.1
ORF158	393	109022	108630	-	hypothetical protein [Salmonella phage GSP001]	99%	4e-90	99.23	UUT40850.1
ORF159	279	109333	109055	-	hypothetical protein bux_13 [Salmonella phage bux]	98%	9e-60	97.83	QIQ61531.1
ORF160	1668	111053	109386	-	DNA transfer protein [Salmonella phage S116]	99%	0.0	100.00	YP_009805270.1
ORF161	237	111399	111163	-	putative A1 protein precursor [Salmonella phage BD13]	93%	6e-30	98.65	UQT65177.1

Table S4. Predicted ORFs in the genome of phage PJN065.

Qeury name	Qeury length	start	stop	strand	Predictive function [Closest hit]	Query Cover	E-values	Identity %	Accession number
ORF1	225	227	3	-	hypothetical protein [Salmonella phage vB_SenS-EnJE1]	100%	1e-28	98.67	YP_010746785.1
ORF2	219	361	579	+	repressor [Salmonella phage NBSal007]	98%	1e-44	100	YP_010746564.1
ORF3	2187	2780	594	-	replicative DNA helicase [Salmonella phage GRNsp50]	99%	0.0	99.45	YP_010748205.1
ORF4	234	1436	2485	-	hypothetical protein [Salmonella enterica]	98%	6e-46	97.4	WP_258082939.1
ORF5	171	3237	3067	-	DNA-binding protein [Salmonella phage vB_StyS-sam]	98%	1e-32	100.0	WP_323471375.1
ORF6	114	4335	4448	+	hypothetical protein QA057_gp07 [Salmonella phage SLMP1]	97%	5e-17	100	YP_010748106.1
ORF7	453	4441	4893	+	hypothetical protein QA058_gp45 [Salmonella phage GRNsp50]	99%	1e-84	94.67	YP_010748201.1
ORF8	204	4890	5093	+	hypothetical protein QA058_gp44 [Salmonella phage GRNsp50]	98%	4e-42	100	YP_010748200.1
ORF9	372	5096	5467	+	hypothetical protein QA028_gp52 [Salmonella phage vB_SenS_TUMS_E	99%	9e-62	94.31	YP_010746371.1

					4]				
ORF10	282	5546	5827	+	holin [Salmonella phage f18SE]	98%	5e-60	100	YP_009191600.1
ORF11	291	5829	6119	+	putative holin [Salmonella phage fmb-p1]	80%	2e-33	98.72	YP_010748223.1
ORF12	489	6097	6585	+	putative endolysin [Salmonella phage fmb-p1]	99%	4e-92	100	YP_010748222.1
ORF13	186	6769	6954	+	hypothetical protein [Salmonella phage SE40]	98%	5e-35	95.08	ARK07400.1
ORF14	198	6951	7148	+	hypothetical protein [Escherichia coli]	98%	4e-23	100	WP_262931455.1
ORF15	159	7145	7303	+	hypothetical protein QCF75_gp30 [Escherichia phage 26]	98%	6e-28	96.15	YP_010749316.1
ORF16	180	7300	7479	+	putative NinZ-like protein [Salmonella phage SS5]	98%	4e-32	94.92	YP_010745747.1
ORF17	225	7622	7846	+	NinH family protein [Salmonella phage SETP3]	98%	1e-46	100	YP_008859658.1
ORF18	123	8026	8148	+	hypothetical protein QA037_gp19 [Salmonella phage vB_SenS_S532]	97%	3e-18	97.5	YP_010746845.1
ORF19	183	9888	10247	+	hypothetical protein QA058_gp34 [Salmonella phage GRNsp50]	98%	2e-33	100	YP_010748190.1
ORF20	132	8388	8519	+	hypothetical protein SaPhSE2_gp29 [Salmonella phage SE2]	97%	2e-22	100	YP_005098125.1

ORF21	507	8548	9054	+	terminase [Salmonella phage vB_SenS-Ent2]	99%	1e-91	98.81	YP_009009925.1
ORF22	1272	9044	10315	+	terminase large subunit [Salmonella phage SLMP1]	99%	0.0	99.76	YP_010748150.1
ORF23	1479	10328	11806	+	putative structural protein [Salmonella phage fmb-p1]	97%	0.0	99.17	YP_010748217.1
ORF24	651	12487	11837	-	amidase [Salmonella phage MA12]	99%	1e-159	99.07	YP_009280131.1
ORF25	1044	12654	13697	+	head morphogenesis protein [Salmonella phage SLMP1]	99%	0.0	98.85	YP_010748147.1
ORF26	459	13700	14158	+	neck whiskers protein [Salmonella phage SLMP1]	99%	1e-85	100	YP_010748146.1
ORF27	120	14312	14431	+	hypothetical protein QA031_gp07 [Salmonella virus VSe103]	97%	2e-18	94.87	YP_010746503.1
ORF28	390	14476	14865	+	hypothetical protein QA057_gp46 [Salmonella phage SLMP1]	99%	5e-89	100	YP_010748145.1
ORF29	702	15056	15757	+	head scaffolding protein [Salmonella phage wksl3]	99%	2e-127	99.57	YP_009608682.1
ORF30	1050	15761	16810	+	major capsid protein [Salmonella phage CKT1]	95%	0.0	99.1	UJP30025.1
ORF31	285	16871	17155	+	head protein [Salmonella phage T102]	98%	4e-41	100	YP_010748282.1

ORF32	351	1716 7	1751 7	+	neck whiskers protein [Salmonella phage SLMP1]	99%	3e-75	100	YP_01074814 0.1
ORF33	189	1755 4	1774 2	+	head-tail joining protein [Salmonella phage SLMP1]	98%	7e-36	100	YP_01074813 9.1
ORF34	510	1774 6	1825 5	+	hypothetical protein QA058_gp19 [Salmonella phage GRNsp50]	99%	9e-119	100	YP_01074817 5.1
ORF35	450	1825 8	1870 7	+	hypothetical protein QA058_gp18 [Salmonella phage GRNsp50]	99%	3e-91	74.13	YP_01074817 4.1
ORF36	360	1870 7	1906 6	+	hypothetical protein QA058_gp17 [Salmonella phage GRNsp50]	99%	1e-79	100	YP_01074817 3.1
ORF37	396	1906 3	1945 8	+	hypothetical protein QA019_gp49 [Salmonella phage SE-W109]	99%	3e-73	98.47	YP_01074581 3.1
ORF38	420	1945 8	1987 7	+	tail protein [Salmonella phage vB_SenS_S532]	99%	2e-96	100	YP_01074686 5.1
ORF39	1170	1987 7	2104 6	+	putative tail protein [Salmonella phage vB_SenS_S532]	99%	0.0	100	YP_01074686 6.1
ORF40	672	2175 0	2107 9	-	putative DNA-binding protein [Salmonella phage GRNsp50]	99%	3e-163	100	YP_01074816 9.1
ORF41	231	2209 4	2186 4	-	hypothetical protein [Salmonella enterica]	98%	3e-48	100	WP_0159849 45.1
ORF42	1155	2331	2216	-	hypothetical	99%	0.0	100	YP_01074816

		8	4		protein QA058_gp11 [Salmonella phage GRNsp50]				7.1
ORF43	330	2371 0	2338 1	-	hypothetical protein QA058_gp10 [Salmonella phage GRNsp50]	99%	3e-73	100	YP_01074816 6.1
ORF44	417	2388 8	2430 4	+	hypothetical protein QA058_gp09 [Salmonella phage GRNsp50]	99%	4e-98	100	YP_01074816 5.1
ORF45	336	2433 1	2466 6	+	hypothetical protein QA058_gp08 [Salmonella phage GRNsp50]	99%	3e-73	99.1	YP_01074816 4.1
ORF46	2280	2465 6	2693 5	+	tail tape measure [Salmonella phage GRNsp50]	99%	0.0	99.6	YP_01074816 3.1
ORF47	501	2693 7	2743 7	+	hypothetical protein QA025_gp28 [Salmonella virus VSe101]	99%	2e-120	100	YP_01074615 9.1
ORF48	516	2743 4	2794 9	+	minor tail protein [Salmonella phage SETP3]	99%	2e-122	100	YP_00111080 1.1
ORF49	366	2794 6	2831 1	+	hypothetical protein QA058_gp04 [Salmonella phage GRNsp50]	99%	2e-85	100	YP_01074816 0.1
ORF50	2559	2830 2	3086 0	+	tail fiber protein [Salmonella phage T102]	99%	0.0	99.41	YP_01074830 1.1
ORF51	2055	3087 3	3292 7	+	tail spike protein [Salmonella phage SLMP1]	99%	0.0	97.51	YP_01074812 1.1
ORF52	93	3304 9	3295 7	-	hypothetical protein			-	-
ORF53	162	3320	3304	-	hypothetical	98%	3e-28	100	YP_01074812

		7	6		protein QA057_gp21 [Salmonella phage SLMP1]				0.1
ORF54	492	33698	33207	-	HNH endonuclease [Salmonella phage SLMP1]	99%	3e-116	99.39	YP_010748119.1
ORF55	2382	36076	33695	-	DNA helicase [Salmonella phage ST3]	99%	0.0	98.36	ASZ77941.1
ORF56	192	36353	36162	-	hypothetical protein QA042_gp04 [Salmonella phage vB_SalS-S10]	98%	6e-36	98.41	YP_010747161.1
ORF57	288	36672	36385	-	virus-type replication repair nuclease [Salmonella phage Kenya-K22]	98%	3e-60	95.79	WCZ56732.1
ORF58	498	37159	36662	-	HNH endonuclease [Salmonella phage SETP3]	99%	3e-120	99.39	YP_001110809.2
ORF59	132	37373	37242	-	hypothetical protein BOX12_gp39 [Salmonella phage BPS11Q3]	97%	1e-20	100	YP_009322846.1
ORF60	2271	39633	37363	-	DNA polymerase I [Salmonella phage F118P13]	99%	0.0	100	YP_010747153.1
ORF61	627	40317	39691	-	TPA: DUF2815 family protein [Salmonella enterica subsp. enterica serovar Enteritidis]	90%	2e-136	100	HCR9424604.1
ORF62	177	40575	40399	-	hypothetical protein QA058_gp53 [Salmonella phage GRNsp50]	98%	9e-33	100	YP_010748209.1

ORF63	1428	41999	40572	-	hypothetical protein QA058_gp52 [Salmonella phage GRNsp50]	99%	0.0	97.47	YP_010748208.1
ORF64	285	42325	42041	-	hypothetical protein BPKS7gp36 [Salmonella phage SS3e]	72%	3e-40	100	YP_224058.1

Table S5. The distribution of tRNA in bacteriophage PJN042.

start	stop	strand	function
25509	25437	-	tRNA-Met-CAT
25682	25609	-	tRNA-Ile-GAT
26332	26261	-	tRNA-Thr-TGT
26811	26740	-	tRNA-Gly-TCC
26894	26822	-	tRNA-Gln-TTG
26976	26904	-	tRNA-Gln-CTG
27283	27212	-	tRNA-Arg-ACG
28402	28325	-	tRNA-Leu-TAG
28723	28651	-	tRNA-Ala-TGC
29521	29451	-	tRNA-Val-TAC
30068	29993	-	tRNA-Lys-TTT
30334	30260	-	tRNA-Met-CAT
30418	30344	-	tRNA-Pro-TGG
30804	30734	-	tRNA-Gly-GCC
31078	31006	-	tRNA-Lys-CTT
32128	32057	-	tRNA-Cys-GCA
32986	32913	-	tRNA-Glu-TTC
33084	32997	-	tRNA-Pseudo-GTA
34074	34001	-	tRNA-Leu-TAA
34902	34828	-	tRNA-Met-CAT
34996	34911	-	tRNA-Ser-GCT
39038	38967	-	tRNA-Arg-TCT

Table S6. Results of significance analysis on 24 h OD_{600} values between phage cocktail and single phage against *S.* typhimurium 015 strain based on bacteriostatic curve.

Phage cocktail	Tukey's multiple comparisons test	Mean diff	95% CI of diff	Adjusted P value
C7	C7 vs. PJN065	-0.2684	-0.2857 to -0.2512	< 0.0001
	C7 vs. PJN042	-0.1156	-0.1329 to -0.09835	< 0.0001
	C7 vs. PJN025	-0.2300	-0.2473 to -0.2127	< 0.0001
	C7 vs. PJN012	-0.02638	-0.04365 to -0.009106	< 0.0001
	C7 vs. vB_SalS_JNS02	-0.2418	-0.2591 to -0.2245	< 0.0001
C18	C18 vs. PJN065	-0.2919	-0.3092 to -0.2746	< 0.0001
	C18 vs. PJN042	-0.1391	-0.1564 to -0.1218	< 0.0001
	C18 vs. PJN025	-0.2535	-0.2708 to -0.2362	< 0.0001
	C18 vs. PJN012	-0.04984	-0.06711 to -0.03257	< 0.0001
	C18 vs. vB_SalS_JNS02	-0.2652	-0.2825 to -0.2480	< 0.0001
C21	C21 vs. PJN065	-0.2766	-0.2939 to -0.2594	< 0.0001
	C21 vs. PJN042	-0.1238	-0.1411 to -0.1065	< 0.0001
	C21 vs. PJN025	-0.2382	-0.2555 to -0.2209	< 0.0001
	C21 vs. PJN012	-0.03458	-0.05185 to -0.01731	< 0.0001
	C21 vs. vB_SalS_JNS02	-0.2500	-0.2673 to -0.2327	< 0.0001
C26	C26 vs. PJN065	-0.2595	-0.2768 to -0.2423	< 0.0001
	C26 vs. PJN042	-0.1067	-0.1240 to -0.08944	< 0.0001
	C26 vs. PJN025	-0.2211	-0.2384 to -0.2038	< 0.0001
	C26 vs. PJN012	-0.01747	-0.03474 to -0.0001963	0.0432
	C26 vs. vB_SalS_JNS02	-0.2329	-0.2501 to -0.2156	<0.0001

Table S7. Results of significance analysis on 24 h OD_{600} values between phage cocktail and single phage against *S.* enteritidis 024 strain based on bacteriostatic curve.

Phage cocktail	Tukey's multiple comparisons test	Mean diff	95% CI of diff	Adjusted P value
C9	C9 vs. PJN065	0.02790	-0.005211 to 0.06101	0.4149
	C9 vs. PJN042	0.04249	0.009383 to 0.07560	0.0003
	C9 vs. PJN025	0.06419	0.03108 to 0.09730	< 0.0001
	C9 vs. PJN012	0.06389	0.03078 to 0.09700	< 0.0001
	C9 vs. vB_SalS_JNS02	-0.06348	-0.09659 to -0.03037	< 0.0001
C20	C20 vs. PJN065	0.05254	0.01943 to 0.08565	< 0.0001
	C20 vs. PJN042	0.06713	0.03402 to 0.1002	< 0.0001
	C20 vs. PJN025	0.08883	0.05572 to 0.1219	< 0.0001
	C20 vs. PJN012	0.08853	0.05542 to 0.1216	< 0.0001
	C20 vs. vB_SalS_JNS02	-0.03884	-0.07195 to -0.005729	0.0026
C25	C25 vs. PJN065	0.02221	-0.01090 to 0.05532	0.9908
	C25 vs. PJN042	0.03681	0.003696 to 0.06992	0.0078
	C25 vs. PJN025	0.05850	0.02539 to 0.09161	< 0.0001
	C25 vs. PJN012	0.05820	0.02509 to 0.09131	< 0.0001
	C25 vs. vB_SalS_JNS02	-0.06917	-0.1023 to -0.03606	< 0.0001
C26	C26 vs. PJN065	-0.009407	-0.04252 to 0.02370	> 0.9999
	C26 vs. PJN042	0.005187	-0.02792 to 0.03830	> 0.9999
	C26 vs. PJN025	0.02688	-0.006231 to 0.05999	0.5573
	C26 vs. PJN012	0.02658	-0.006531 to 0.05969	0.6012
	C26 vs. vB_SalS_JNS02	-0.1008	-0.1339 to -0.06768	< 0.0001
